# Restoring the epigenetically silenced lncRNA COL18A1-AS1 represses ccRCC progression by lipid browning via miR-1286/KLF12 axis

**DOI:** 10.1038/s41419-022-04996-2

**Published:** 2022-07-04

**Authors:** Yuenan Liu, Jun Wang, Yi Shou, Wenjie Xu, Ziwei Huang, Jiaju Xu, Kailei Chen, Jingchong Liu, Di Liu, Huageng Liang, Hongmei Yang, Xiaoping Zhang

**Affiliations:** 1 Department of Urology, Union Hospital, Tongji Medical College, Huazhong University of Science and Technology, Wuhan, 430022 P. R. China; 2 Institute of Urology, Tongji Medical College, Huazhong University of Science and Technology, Wuhan, 430022 P. R. China; 3 Department of Breast and Thyroid Surgery, Union Hospital, Tongji Medical College, Huazhong University of Science and Technology, Wuhan, 430022 P. R. China; 4 Department of Pathogenic Biology, School of Basic Medicine, Huazhong University of Science and Technology, Wuhan, 430030 P. R. China; 5 Shenzhen Huazhong University of Science and Technology Research Institute, Shenzhen, 518000 P. R. China

**Keywords:** Renal cell carcinoma, Prognostic markers, RNA

## Abstract

Abnormal accumulation of lipids has been highlighted in the progression of clear cell renal cell carcinoma (ccRCC). However, the underlying mechanism remains unclear. Emerging evidence suggests long noncoding RNAs (lncRNAs) participate in the regulation of lipid metabolism. In this study, we found lncRNA COL18A1-AS1 was downregulated in ccRCC and that higher COL18A1-AS1 expression indicated better prognosis. Decreased COL18A1-AS1 expression was caused by DNA methylation at the CpG islands within its promoter. Restoring the epigenetically silenced COL18A1-AS1 repressed tumor progression, promoted lipid browning and consumption in vitro and in vivo. Mechanistically, COL18A1-AS1 could competitively bind miR-1286 to increase the expression of Krüppel-like factor 12 (KLF12). Downregulation of COL18A1-AS1 in ccRCC resulted in the low expression of KLF12. COL18A1-AS1/KLF12 positively regulated uncoupling protein 1 (UCP1)–mediated lipid browning, which promotes tumor cell “slimming” and inhibits tumor progression. When tumor cell “slimming” occurred, lipid droplets turned into tiny pieces, and lipids were consumed without producing ATP energy. Taken together, our findings on COL18A1-AS1-miR-1286/KLF12 axis revealed a potential mechanism of abnormal accumulation of lipids in ccRCC and could be a promising therapeutic target for ccRCC patients.

## Introduction

Renal cell carcinoma (RCC) is one of the most common and lethal malignant tumors in the urinary system [1, 2]. Clear cell renal cell carcinoma (ccRCC) is the most prevalent pathological subtype, account for 70–80% of RCCs [3]. It is estimated that there will be 76,080 new cases and 13,780 mortalities of RCC in the USA in 2021 [4]. Metabolic abnormalities are regarded as one of the characteristics of tumor cells [5]. For ccRCC, lipid metabolism dysfunction could be the most prominent feature. Morphologically, ccRCC cells are distinguished by aberrant lipid accumulation in the cytoplasm, which protects cells and promotes tumor progression [6]. Furthermore, studies showed that RCC was a metabolic disorder [7], and atherosclerosis and diabetes increased the risk of RCC [8, 9]. However, the molecular mechanism remains unclear.

Adipose tissue includes white adipose tissue (WAT) and brown adipose tissue (BAT). WAT mainly functions as energy storage, while BAT is responsible for heat generation [10]. Lipid browning is a process by which WAT transforms into BAT. In this process, lipids were consumed through thermogenesis without additional adenosine triphosphate (ATP) production [11]. Our previous studies concentrated on the application of lipid browning in ccRCC and proposed the new concept tumor cell “slimming” [12, 13]. By tumor cell “slimming”, lipid droplets turn into tiny pieces and tumor cell is reduced in volume, which leads to suppression of tumor progression. Uncoupling protein 1 (UCP1) mediated the lipid browning and was regarded as a hallmark of BAT [14]. Melatonin was revealed to increase the expression of PGC1A and UCP1, which activated lipid browning and repressed ccRCC progression [12]. Moreover, Phospholipase C-like 1 (PLCL1) was identified to activate lipid browning via stabilizing the protein of UCP1, which leaded to the inhibition of ccRCC [13]. Krüppel-like factor 12 (KLF12) was a transcription factor that regulates gene expression during vertebrate development and carcinogenesis [15]. KLF12 plays different roles in different types of cancers. In ovarian cancer, KLF12 inhibited anoikis resistance and functioned as a tumor suppressor [16]. Conversely, KLF12 could also promote stemness features of pancreatic cancer cells [17]. However, what role KLF12 plays in ccRCC remains unclear. Besides, it has been reported that KLF12 is associated with preadipocyte differentiation and lipid accumulation [18]. But few studies about the relationship between KLF12 and lipid metabolism and lipid browning were reported in tumor cells.

Long noncoding RNAs (lncRNAs) are ubiquitously involved in various biological processes of tumor cells [19]. Numerous studies focused on their roles in metabolism regulation, including lipid metabolism [20]. Study reported that lncRNA TINCR promoted de novo lipid biosynthesis in nasopharyngeal carcinoma [21]. Also, lncRNA LINC00473 was identified to be correlated with UCP1 and promoted the thermogenic adipocyte development [22]. However, the roles of lncRNA in abnormal accumulation of lipids in ccRCC remains unclear. COL18A1 Antisense RNA 1 (COL18A1-AS1), is a lncRNA located in 21q22.3. Some bioinformatics analysis has reported that COL18A1-AS1 could be a biomarker for Cholangiocarcinoma [23, 24] and RCC [25, 26]. Gene Set Enrichment Analysis (GSEA) showed that COL18A1-AS1 might participated in lipid metabolism signaling pathway. Therefore, we explored the potential mechanism of COL18A1-AS1 in ccRCC.

In this study, we screened the differentially expressed lncRNAs in ccRCC tissues and identified COL18A1-AS1 as a novel tumor suppressor. The relatively low expression of COL18A1-AS1 in ccRCC was the result of DNA hypermethylation of its promoter region. Overexpression of COL18A1-AS1 suppressed lipids accumulation and inhibited ccRCC cells proliferation and metastasis. In addition, COL18A1-AS1 was a molecular sponge of miR-1286 to increase the expression of KLF12, which finally regulated the UCP1-mediated lipid browning. These findings might provide a novel biomarker and potential therapeutic target for ccRCC.

## Results

### COL18A1-AS1 was downregulated and could be a prognostic biomarker for ccRCC

**Fig. 1:**
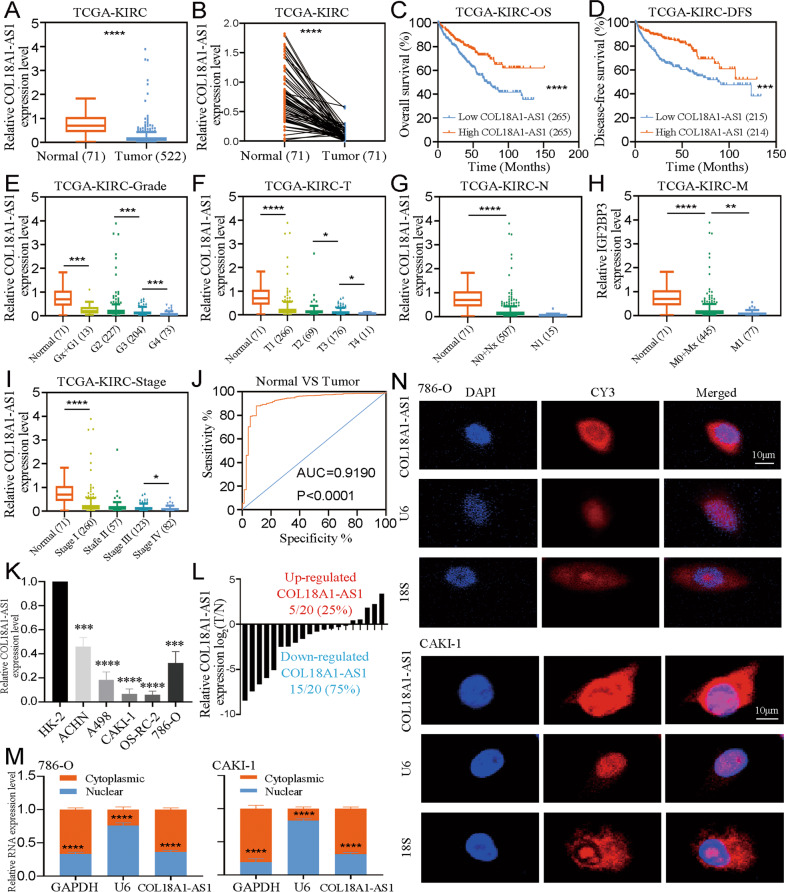
COL18A1-AS1 was downregulated and could be a biomarker for ccRCC. **A** The expression level of COL18A1-AS1 in 72 normal renal tissues and 522 ccRCC tissues from TCGA database. **B** The expression level of COL18A1-AS1 in 71 pairs of matched ccRCC and normal tissues from TCGA database. **C, D** The Kaplan–Meier curves of COL18A1-AS1 in ccRCC for overall survival and disease-free survival. **E**–**I** The expression of COL18A1-AS1 was related with various clinicopathological factors. **J** The receiver operating characteristic (ROC) curves of COL18A1-AS1 (AUC = 0.9190; *p* < 0.0001). **K** The expression level of COL18A1-AS1 in RCC cell lines (ACHN, A498, CAKI-1, OS-RC-2, and 786-O) and normal cell line (HK-2). **L** The expression level of COL18A1-AS1 in 20 ccRCC tissues and adjacent normal tissues. **M** Localization of COL18A1-AS1 was assessed by PCR in CAKI-1 and 786-O cells. U6 and GAPDH were used as positive controls for nuclear RNA and cytoplasmic RNA, respectively. **N** The distribution of COL18A1-AS1 was analyzed by FISH in CAKI-1 and 786-O cells. 18 S and U6 showed cytoplasm and nucleus, respectively. Scale bars, 10 μm. *P < 0.05, **P < 0.01, ***P < 0.001, ****P < 0.0001. Error bars indicate mean ± SD. All the experiments were performed in triplicate.

To explore the aberrantly expressed lncRNAs in ccRCC, we sequenced 3 pairs of ccRCC tissues and adjacent normal tissues. COL18A1-AS1 was one of the most obviously altered lncRNAs (Supplementary Fig. 1). Previous studies identified COL18A1-AS1 could be a biomarker for ccRCC, but its roles remain unclear [25, 26]. Therefore, we focused on COL18A1-AS1. From TCGA-KIRC, we found COL18A1-AS1 was obviously downregulated in ccRCC (Fig. 1A, B). And COL18A1-AS1 expression was an independent protective factor of ccRCC (Supplementary Table 1). Kaplan–Meier analysis showed ccRCC patients with higher COL18A1-AS1 expression had a better overall survival (OS) (Fig. 1C, Supplementary Fig. 2) and disease-free survival (DFS) (Fig. 1D, Supplementary Fig. 3). Furthermore, CO18A1-AS1 expression was also associated with tumor grade and stage (Table 1, Fig. 1E–I). The receiver operating characteristics (ROC) curve also revealed COL18A1-AS1 had a good diagnostic value (Fig. 1J).

**Table 1:** Correlation between COL18A1-AS1 expression and clinicopathological parameters of ccRCC patients.

Parameter		Number	COL18A1-AS1 expression		*P*-value
			Low (*n* = 261)	High (*n* = 261)	
Age (years)	<60	244	133	111	0.054
	≥60	278	128	150	
Gender	Female	180	103	77	0.017
	Male	342	158	184	
T stage	T1 or T2	335	194	141	<0.001
	T3 or T4	187	67	120	
N stage	N0 or NX	507	255	252	0.432
	N1	15	6	9	
M stage	M0 or MX	445	242	203	<0.001
	M1	77	19	58	
G grade	G1 or G2orGx	245	148	97	<0.001
	G3 or G4	277	113	164	
TNM stage	I + II	317	190	127	<0.001
	III + IV	205	71	134	

*TNM* tumor-node-metastasis, *COL18A1-AS1* COL18A1 Antisense RNA 1, *ccRCC* clear cell renal cell carcinoma.

Next, qRT-PCR showed that COL18A1-AS1 was downregulated in RCC cells (Fig. 1K). Meanwhile, 75% of ccRCC tissues expressed COL18A1-AS1 lower than matched normal tissues (Fig. 1L). RNA cellular fraction assay showed that COL18A1-AS1 mainly located in cytoplasm of ccRCC cells (Fig. 1M). And FISH assays also exhibited similar results (Fig. 1N). Above all, we discovered that cytoplasm-located lncRNA COL18A1-AS1 was downregulated in ccRCC and could be a prognostic biomarker.

### COL18A1-AS1 repressed ccRCC progression and eliminated lipids accumulation in vitro

**Fig. 2:**
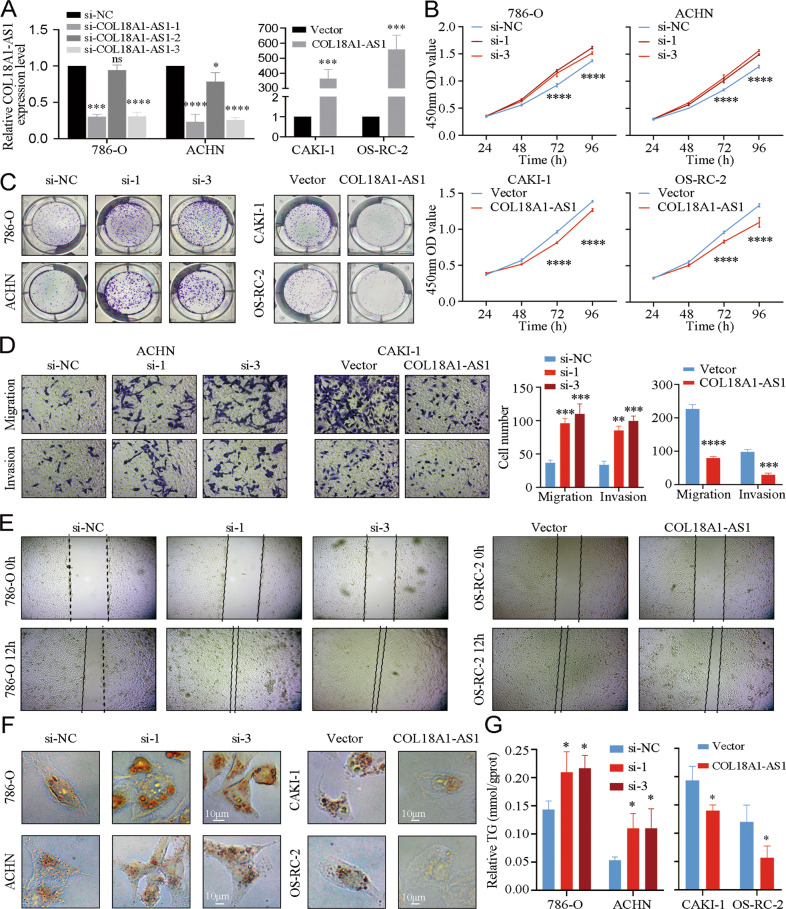
COL18A1-AS1 repressed ccRCC progression and eliminated lipids accumulation in vitro. **A** qRT-PCR assays were applied to analyze the expression level of COL18A1-AS1 after transfection by si-RNA or COL18A1-AS1 overexpression vector for 24 h in RCC cells. **B**, **C** Cell proliferation ability of RCC cells after knocking down or overexpressing COL18A1-AS1 was determined using CCK8 assays or colony formation assays. **D**, **E** Cell migration and invasion ability of RCC cells was measured with Transwell assays or wound healing assays (Magnification: ×100 for Transwell assays and x40 for wound healing assays). **F** Photomicrographs of Oil Red staining of RCC cells knocked down or overexpressed COL18A1-AS1 compared with the negative control. Scale bars, 10 μm. **G** Relative TG (mmol/gprot) levels in RCC cells with COL18A1-AS1 overexpression or knockdown assessed by a triglyceride assay kit. *P < 0.05, **P < 0.01, ***P < 0.001, ****P < 0.0001. Error bars indicate mean ± SD. All the experiments were performed in triplicate.

To explore the roles of COL18A1-AS1 in ccRCC, GSEA based on GEO dataset (GSE53757) was performed. Free fat acid oxidation, tumorigenesis, proliferation, and epithelial to mesenchymal transition were enriched in low COL18A1-AS1 expression group (Supplementary Fig. 4A). In RCC cell lines, ACHN and 786-O expressed COL18A1-AS1 relatively high, thus we used si-RNA to knock it down. CAKI-1 and OS-RC-2 expressed COL18A1-AS1 relatively low, so we constructed overexpression vector and transfected these cells with it (Fig. 2A). Also, we examined the mRNA and protein expression levels of COL18A1 in both COL18A1-AS1 knockdown and overexpression cells, to confirm the phenotype is only due to COL18AA1-AS1 (Supplementary Fig. 4B, C). CCK8 assays and colony formation analysis demonstrated that COL18A1-AS1 tended to suppress the proliferation ability of RCC cells (Fig. 2B, C). Next, Transwell assays and wound healing assays suggested knocking down COL18A1-AS1 promoted migration and invasion of cells, and overexpressing COL18A1-AS1 repressed tumor cell metastasis (Fig. 2D, E and Supplementary Fig. 4D, E). Then, oil red staining assays showed more lipids accumulated in cells with COL18A1-AS1 knockdown. And lipids were consumed and lipid droplets transformed into small pieces in COL18A1-AS1 overexpressed cells (Fig. 2F). And the triglyceride detection assays found a negative correlation between COL18A1-AS1 expression and triglyceride content in RCC cells (Fig. 2G). To sum up, COL18A1-AS1 eliminated lipids accumulation and repressed ccRCC progression in vitro.

### DNA methylation leaded to the low expression of COL18A1-AS1 in ccRCC

**Fig. 3:**
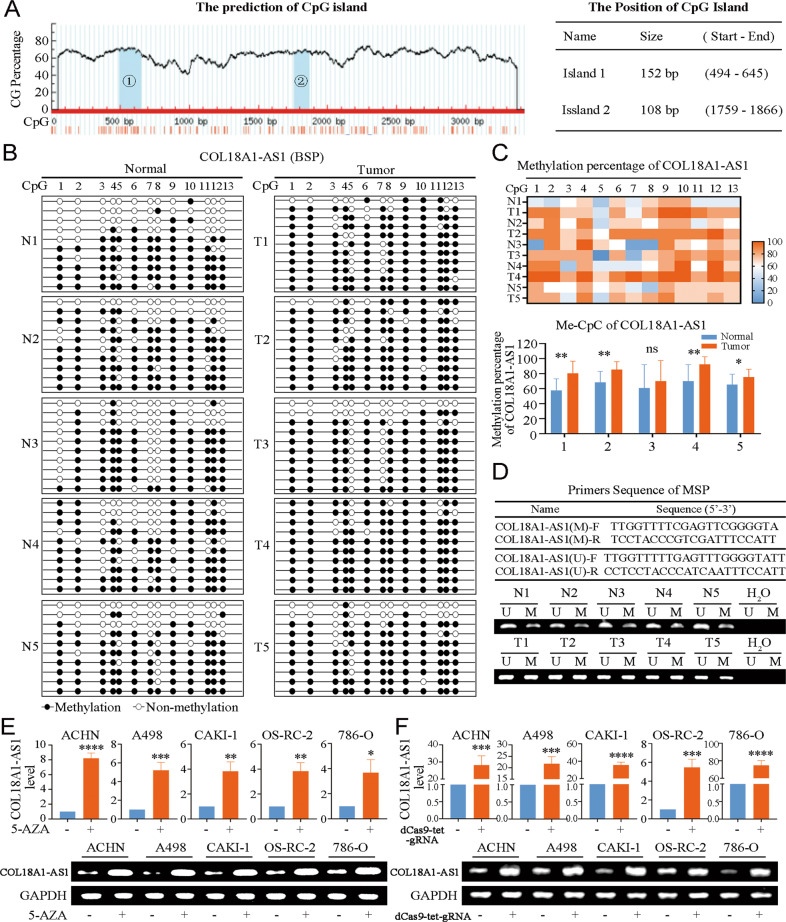
DNA methylation caused the low expression of COL18A1-AS1 in ccRCC. **A** Prediction analysis of CpG islands in the sequence range of 3400 bp upstream from the transcriptional start site in the COL18A1-AS1 promoter region. **B** BSP results of COL18A1-AS1 methylation status in adjacent tissues and ccRCC tissues. **C** Heat map of methylation percentage of COL18A1-AS1 and histogram of quantitative results. **D** The primers sequence of MSP and representative MSP results of COL18A1-AS1 methylation status in adjacent tissues and ccRCC tissues. **E** The expression levels of COL18A1-AS1 in RCC cell lines after 5-AZA treatment. **F** The expression levels of COL18A1-AS1 in RCC cell lines after CRISPR/dCas9-mediated editing system specific demethylation. *P < 0.05, **P < 0.01, ***P < 0.001, ****P < 0.0001. Error bars indicate mean ± SD. All the experiments were performed in triplicate.

DNA methylation is one of the most common epigenetic modifications, which silences gene expression [27]. To explore the mechanism of low expression of CO18A1-AS1 in ccRCC, we analyzed the methylation level of its promoter CpG island. From cBioPortal database, we found COL18A1-AS1 expression was negatively correlated with DNA methylation status (Supplementary Fig. 5A). In TCGA-KIRC, 8 CpG sites were identified. The methylation levels of 5 CpG sites were significantly higher in ccRCC (Supplementary Fig. 5B). Interestingly, we found these five CpG sites were significantly negatively correlated with COL18A1-AS1 expression (Supplementary Fig. 5C–J). Next, we identified CpG islands in the promoter of COL18A1-AS1 through MethPrimer tool (Fig. 3A). Bisulfite sequencing PCR (BSP) was performed on 5 pairs of ccRCC and matched normal tissues. 13 CpG sites were identified (Fig. 3B, C and Supplementary Fig. 5K). The methylation levels of 4 ccRCC were significantly higher than matched normal tissues. Then, we applied Methylation-Specific PCR (MSP) to confirm these results. The MSP sequence was showed in Supplementary Fig. 5L. We found COL18A1-AS1 promoter was obviously hypermethylated in ccRCC tissues (Fig. 3D). Cancer Cell Line Encyclopedia database provided the methylation level of COL18A1-AS1 in RCC cell lines. Methylation was enriched in COL18A1-AS1 promoter region in RCC cell lines (Supplementary Fig. 5M). 5-AZA is a demethylating drug. After 24 hours of 5-AZA treatment, COL18A1-AS1 expression level was detected with qRT-PCR. We found 5-AZA significantly increased the expression of COL18A1-AS1 (Fig. 3E). To establish a solid association of COL18A1-AS1 methylation with its expression level, a CRISPR/dCas9-mediated editing system for COL18A1-AS1 specific demethylation was constructed (Supplementary Fig. 6). These resulted revealed that specific demethylation significantly promoted the expression of COL18A1-AS1 (Fig. 3F). Therefore, we suggested DNA methylation caused the low expression of COL18A1-AS1 in ccRCC.

### COL18A1-AS1 interacted with miR-1286 in ccRCC

**Fig. 4:**
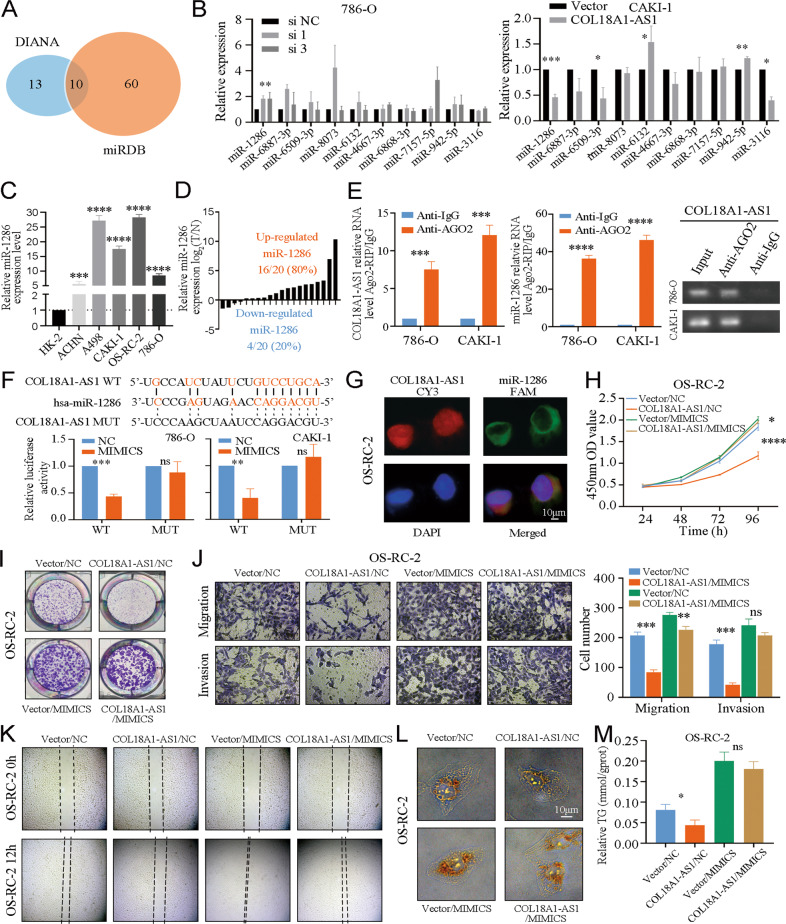
COL18A1-AS1 interacted with miR-1286 in ccRCC. **A** A Venn diagram of two independent miRNA prediction analysis. Blue: DIANA database. Orange: miRDB database. **B** qRT-PCR results of expression level of candidate miRNAs in 786-O and CAKI-1 cells transfected with si-COL18A1-AS1 or COL18A1-AS1 vector. **C** The expression level of miR-1286 in RCC cell lines (ACHN, A498, CAKI-1, OS-RC-2, and 786-O) and normal cell line (HK-2). **D** The expression level of miR-1286 in 20 ccRCC tissues and adjacent normal tissues. **E** RIP assays showed the abundance of COL18A1-AS1 and miR-1286 in AGO2 complex in CAKI-1 and 786-O cells. **F** Luciferase reporter assays detected the luciferase activity of COL18A1-AS1-WT or COL18A1-AS1-Mut under miR-1286 MIMICS in CAKI-1 and 786-O cells. **G** Representative FISH images showed the co-localization of COL18A1-AS1 (red) and miR-1286 (green) in the cytoplasm of 786-O cells. Cell nuclear appear in blue (DAPI). Scale bars, 10 μm. **H**, **I** Cell proliferation ability of OS-RC-2 cells co-transfected with COL18A1-AS1 vector and/or miR-1286 MIMICS was determined using CCK8 assays or colony formation assays. **J**, **K** Cell migration and invasion ability of OS-RC-2 cells was measured with Transwell assays or wound healing assays (Magnification: ×100 for Transwell assays and x40 for wound healing assays). **L** Photomicrographs of Oil Red staining of OS-RC-2 cells. Scale bars, 10 μm. **M** Relative TG (mmol/gprot) levels in OS-RC-2 cells assessed by a triglyceride assay kit. *P < 0.05, **P < 0.01, ***P < 0.001, ****P < 0.0001. Error bars indicate mean ± SD. All the experiments were performed in triplicate.

Emerging evidences have shown lncRNAs interact with miRNAs and work as ceRNAs [28]. Therefore, miRDB and DIANA-LncBase were applied to identify 10 miRNAs predicted to bind COL18A1-AS1 (Fig. 4A). Next, we knocked down or overexpressed COL18A1-AS1 in cells. We found only miR-1286 expression changed significantly (Fig. 4B). miR-1286 was upregulated both in RCC cells (Fig. 4C) and ccRCC tissues (Fig. 4D). Furthermore, RNA-binding protein immunoprecipitation (RIP) was conducted to validate that COL18A1-AS1 and miR-1286 were enriched by anti-AGO2 antibody in 786-O and CAKI-1 cells (Fig. 4E). And luciferase activity has significantly weakened in RCC cells transfected with miR-1286 MIMICS and COL18A1-AS1 wild-type (WT) vector (Fig. 4F). Then, FISH revealed that COL18A1-AS1 and miR-1286 were co-located in cytoplasm of ccRCC cells (Fig. 4G and Supplementary Fig. 6G).

After that, we designed rescue experiments to study the function of miR-1286. We found the decrease of proliferation by overexpression of COL18A1-AS1 could be rescue by miR-1286 MIMICS (Fig. 4H, I and Supplementary Fig. 7A, B). And restoration of miR-1286 repaired the migration and invasion ability suppressed by upregulation of COL18A1-AS1 in ccRCC cells (Fig. 4J, K and Supplementary Fig. 7C, D). We also found transfection of miR-1286 MIMICS increased the lipid droplets and total TG levels in ccRCC cells (Fig. 4L, M and Supplementary Fig. 7E, F). Overall, we demonstrated that COL18A1-AS1 interacted with miR-1286 and exerted its roles via miR-1286 in ccRCC.

### COL18A1-AS1 regulated the expression of KLF12 through miR-1286 in ccRCC

**Fig. 5:**
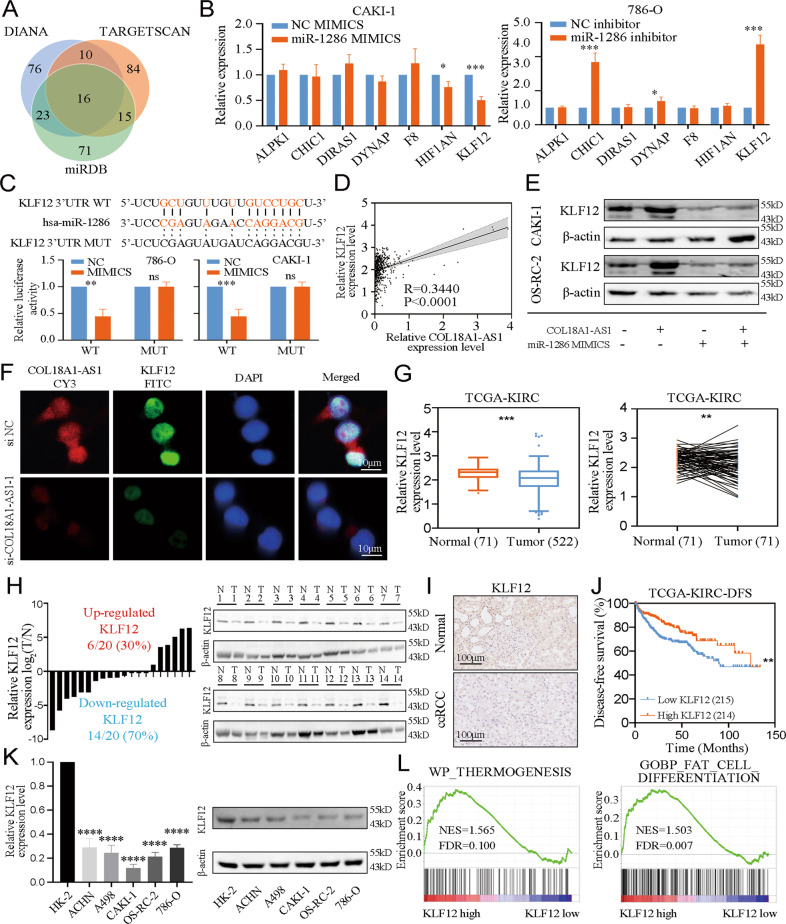
COL18A1-AS1 regulated the expression of KLF12 through competitively binding with miR-1286 in ccRCC. **A** A Venn diagram of three independent miRNA prediction analysis. Blue: DIANA database. Orange: TARGETSCAN database. Green: miRDB database. **B** qRT-PCR results of expression level of candidate mRNAs in 786-O and CAKI-1 cells transfected with miR-1286 MIMICS or inhibitor. **C** Luciferase reporter assays detected the luciferase activity of KLF12 3'UTR-WT or KLF12 3'UTR-Mut under miR-1286 MIMICS in CAKI-1 and 786-O cells. **D** Positive correlation between COL18A1-AS1 and KLF12 expression in ccRCC tissues from TCGA database. **E** Western blotting analysis of KLF12 protein in CAKI-1 and OS-RC-2 cells co-transfected with COL18A1-AS1 vector and/or miR-1286 MIMICS. **F** Representative FISH and IF images showed the expression level of COL18A1-AS1 (red) and KLF12 (green) in 786-O cells. Cell nuclear appear in blue (DAPI). Scale bars, 10 μm. **G** The expression level of KLF12 in normal renal and ccRCC tissues from TCGA database (unpaired or paired). **H** qRT-PCR and Western blotting assays showed the expression level of KLF12 in ccRCC tissues and adjacent normal tissues. **I** IHC for KLF12 in ccRCC tissues and paired normal tissues. Scale bars, 100 μm. **J** The Kaplan–Meier curve of KLF12 in ccRCC for disease-free survival. **K** qRT-PCR and Western blotting assays showed the expression level of KLF12 in RCC cell lines (ACHN, A498, CAKI-1, OS-RC-2 and 786-O) and normal cell line (HK-2). **L** GSEA for the correlations between the thermogenesis and fat cell differentiation signaling pathway in ccRCC with the levels of the KLF12 mRNA, according to GEO database (GSE53757). FDR < 0.25 and *P* < 0.05 were considered statistically significant. *P < 0.05, **P < 0.01, ***P < 0.001, ****P < 0.0001. Error bars indicate mean ± SD. All the experiments were performed in triplicate.

Next, DIANA, TARGETSCAN, and miRDB were used to screen the target genes of miR-1286 (Fig. 5A). Kaplan–Meier survival analysis showed among 16 candidate genes, only 7 genes were associated with prognosis of ccRCC (Supplementary Fig. 8). Next, we transfected miR-1286 MIMICS or inhibitor in cells. Only KLF12 expression showed significant changes (Fig. 5B). And luciferase activity has significantly weakened in RCC cells transfected with miR-1286 MIMICS and KLF12 mRNA 3'UTR WT vector (Fig. 5C). Moreover, a positive correlation was showed between COL18A1-AS1 and KLF12 in TCGA-KIRC (Fig. 5D). Western blotting showed KLF12 expression was controlled by COL18A1-AS1 and miR-1286 (Fig. 5E and Supplementary Fig. 9A). FISH and IF assays also demonstrated KLF12 was regulated by COL18A1-AS1 (Fig. 5F and Supplementary Fig. 9B). These results revealed that KLF12 might be the downstream target of COL18A1-AS1.

KLF12 is a member of KLF (Krüppel-like factor) family, which is a DNA-binding transcriptional regulator [29]. We found KLF12 was significantly downregulated in ccRCC tissues (Fig. 5G–I) and that high KLF12 expression indicated a better DFS (Fig. 5J). And KLF12 expression was lower in RCC cells than in HK-2 cells (Fig. 5K). GSEA exhibited that KLF12 was correlated with thermogenesis and fat cell differentiation (Fig. 5L). Therefore, we validated COL18A1-AS1 regulated KLF12 through competitively binding with miR-1286 in ccRCC.

### COL18A1-AS1/KLF12 axis repressed ccRCC progression through UCP1-mediated lipid browning

**Fig. 6:**
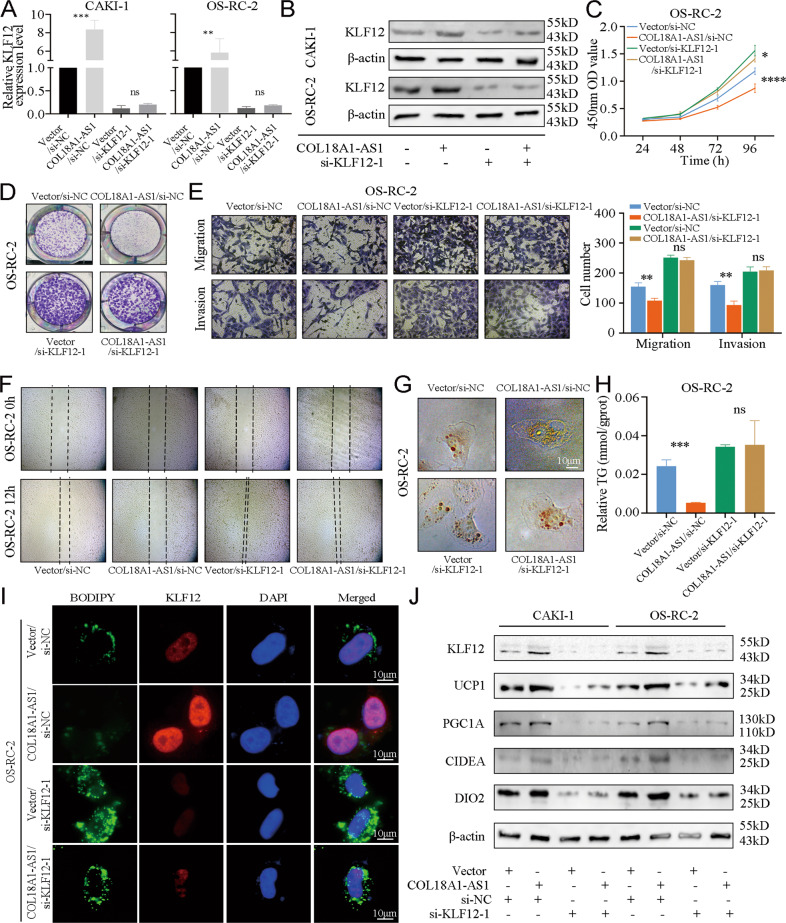
COL18A1-AS1/KLF12 axis repressed ccRCC progression through UCP1-mediated lipid browning. **A**, **B** qRT-PCR and Western blotting assays showed the expression level of KLF12 in CAKI-1 and OS-RC-2 cells co-transfected with COL18A1-AS1 vector and/or si-KLF12. **C**, **D** Cell proliferation ability of OS-RC-2 cells co-transfected with COL18A1-AS1 vector and/or si-KLF12 was determined using CCK8 assays or colony formation assays. **E**, **F** Cell migration and invasion ability of OS-RC-2 cells was measured with Transwell assays or wound healing assays (Magnification: ×100 for Transwell assays and x40 for wound healing assays). **G** Photomicrographs of Oil Red staining of OS-RC-2 cells. Scale bars, 10 μm. **H** Relative TG (mmol/gprot) levels in OS-RC-2 cells assessed by a triglyceride assay kit. **I** Representative photographs of IF staining for lipids with BODIPY 493 of 503 (green) and KLF12 (red) in the cells co-transfected with COL18A1-AS1 vector and/or si-KLF12. Cell nuclear appear in blue (DAPI). Scale bars, 10 μm. **J** Protein levels of lipid browning marker genes (UCP1, PGC1A, CIDEA, and DIO2) in CAKI-1 and OS-RC-2 cells co-transfected with COL18A1-AS1 vector and/or si-KLF12 assessed by western blotting. *P < 0.05, **P < 0.01, ***P < 0.001, ****P < 0.0001. Error bars indicate mean ± SD. All the experiments were performed in triplicate.

We designed a series of rescue experiments to validate the roles of COL18A1-AS1/KLF12 axis in ccRCC progression. We transfected si-KLF12 in ccRCC cells that had been stably overexpressed COL18A1-AS1 with lentivirus (Fig. 6A, B). Next, cell function experiments showed knocking down KLF12 reversed the negative roles of COL18A1-AS1 on the proliferation, migration, and invasion ability of RCC cells (Fig. 6C–F and Supplementary Fig. 9C–F). And transfection of si-KLF12 increased the lipid accumulation and total TG levels in ccRCC cells (Fig. 6G, H and Supplementary Fig. 9G, H). IF also exhibited similar results (Fig. 6I). As KLF12 was involved with thermogenesis and fat cell differentiation, we hypothesized that KLF12 might participate in lipid browning. To verify it we found multiple browning markers were downregulated after KLF12 knocked down (Supplementary Fig. 10A). Western blotting also exhibited downregulation of KLF12 reversed the positive regulation of COL18A1-AS1 on UCP1 and other browning markers in ccRCC (Fig. 6J). Thus, we verified that COL18A1-AS1/KLF12 axis repressed ccRCC progression through UCP1-mediated lipid browning.

### COL18A1-AS1 promoted tumor slimming and repressed ccRCC progression in vivo

**Fig. 7:**
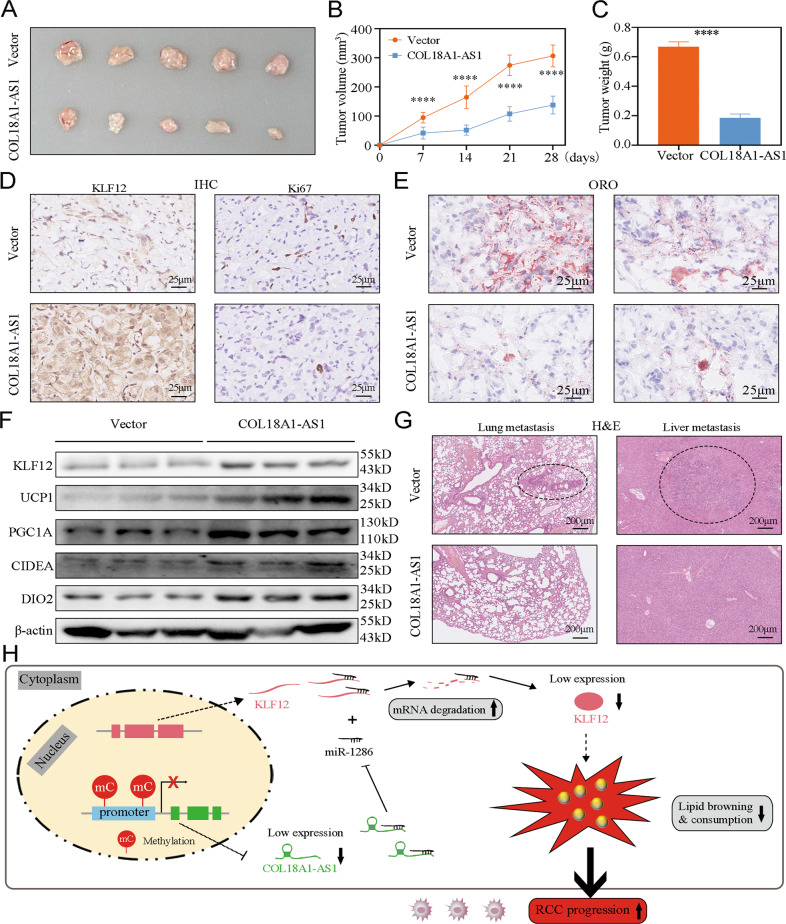
COL18A1-AS1 promoted tumor slimming and repressed ccRCC progression in vivo. **A** OS-RC-2 cells with COL18A1-AS1 stably overexpressed were subcutaneously injected into nude mice. **B**, **C** The volume and weight of xenograft tumors in two groups (*n* = 5). **D** IHC assays were performed to measure KLF12 and the marker of tumor malignancy (Ki67) expression levels in two groups. Scale bars, 25 μm. **E** Oil Red staining of the tumor xenografts from the COL18A1-AS1-overexpressing and control groups. Scale bars, 25 μm. **F** Western blotting assays were performed to measure KLF12 and the markers of lipid browning (UCP1, PGCIA, CIDEA, and DIO2) expression levels in two groups. **G** H&E staining of the lung metastasis and liver metastasis from the COL18A1-AS1-overexpressing and control groups. Scale bars, 200 μm. **H** Schematic diagram illustrated that methylation of the COL18A1-AS1 promoter region in ccRCC inhibited the expression of COL18A1-AS1, thus leaded to lipid abnormal accumulation and RCC progression via miR-1286/KLF12 axis. *P < 0.05, **P < 0.01, ***P < 0.001, ****P < 0.0001. Error bars indicate mean ± SD. All the experiments were performed in triplicate.

Finally, we explored the functions of COL18A1-AS1 in ccRCC in vivo. OS-RC-2 cells with COL18A1-AS1 stable overexpression or negative control were respectively implanted subcutaneously into 4-week-old nude mice to construct xenograft tumor models. We found both the size and weight of tumors were significantly repressed in the COL18A1-AS1 overexpression group (Fig. 7A–C). IHC on subcutaneous xenografts showed KLF12 was upregulated and Ki67 was downregulated in the group overexpressing COL18A1-AS1 (Fig. 7D). Tissues oil red staining exhibited that more lipid accumulated in negative control group (Fig. 7E). The protein levels of lipid browning markers were significantly increased after overexpression COL18A1-AS1 (Fig. 7F). Then, we constructed the metastasis model. We found overexpression of COL18A1-AS1 reduced lung and liver metastasis (Fig. 7G).

In Fig. 7H, we generated a schematic diagram of this process. Decreased COL18A1-AS1 expression was caused by DNA methylation at the CpG islands within its promoter. COL18A1-AS1 could competitively bind miR-1286 to increase the expression of KLF12. Restoring the epigenetically silenced COL18A1-AS1 repressed tumor proliferation and metastasis through promoting lipid browning and consumption in vitro and in vivo.

## Discussion

Abnormal lipid accumulation has been considered as one of the most characteristic features of ccRCC. Emerging evidences suggested ccRCC cells received lipid metabolites from surrounding fat cells [30, 31]. Meanwhile, the catabolism of lipids was reduced in ccRCC, which might result in the abnormal lipid accumulation [32, 33]. However, why ccRCC cell tends to store the lipid drops in it and what roles lipid drops play in tumor cells still remains unclear. In previous study, our group observed the inhibitory effect on ccRCC cells when attempting to consume lipid drops [12, 13]. In this present study, we furtherly identified a novel pathway of lipid browning in ccRCC. We found COL18A1-AS1/KLF12 axis activated the genes related with lipid browning, including UCP1, PCG1A, DIO2, and CIDEA, thus promoted lipid consumption and inhibited ccRCC progression.

In ccRCC, numerous upregulated or downregulated lncRNAs were reported. For example, lncRNA SNHG12 was upregulated and promoted sunitinib resistance in ccRCC [34]. lncRNA lnc-TSI was reported to inhibited invasion and metastasis and could be a potential therapeutic target [35]. However, the functions of lncRNAs in lipid accumulation and consumption of ccRCC are relatively less studied. COL18A1-AS1 was widely studied as a diagnostic and prognostic biomarker of ccRCC [25, 26, 36]. However, the function of COL18A1-AS1 has not been published yet. For cytoplasmic located lncRNA, lncRNA-miRNA interaction is a crucial functional mechanism [37]. For instance, lncRNA MALAT1 interacted with miR-205 and promoted aggressive RCC [38]. In this work, we identified COL18A1-AS1 interacted with miR-1286. Previously, miR-1286 was uncovered to play a carcinogenesis role in lung adenocarcinoma [39], which was similar in our study.

Epigenetic alterations have been regarded as an important and ubiquitous regulation of gene expression in tumorigenesis [40]. DNA methylation, one of the most common epigenetic regulations, is the process that methylation of cytosine residues in the context of CpG dinucleotides of DNA [41]. In previous studies, DNA methylations of different genes were reported to influence the progression of ccRCC. Methylation of PCK2 promoter region repressed its expression, so that cells survival from endoplasmic reticulum stress [42]. Moreover, NELL1 and NELL2 were silenced by hypermethylated, thereby promoting the progression of RCC [43]. However, relatively fewer methylation-regulated lncRNAs have been identified. In this present study, we found COL18A1-AS1 was downregulated in ccRCC. The expression level of COL18A1-AS1 was negative correlated with the methylation level of its promoter region. And 5-AZA increased COL18A1-AS1 expression. Therefore, we uncovered COL18A1-AS1 was silenced by DNA methylation in ccRCC.

Krüppel-like factors (KLFs) are a family of DNA-binding transcriptional regulators that influence diverse physiological and pathological processes [29]. Up to now, 17 KLFs have been identified and some of them was involved with RCC development. KLF4 and KLF5 were downregulated and played tumor suppressor roles in RCC [44, 45]. Interestingly, a KLF6-driven transcriptional network was identified to link lipid homeostasis and tumor growth in RCC [46]. However, fewer studies about the functions of KLF12 in ccRCC were published before. We determined that KLF12 was downregulated in ccRCC. Functionally, KLF12 positively regulated lipid browning marker genes and promoted lipid consumption in RCC cells. Thus, we supposed KLF12 was a tumor suppressor and related with lipid metabolism in ccRCC. However, the molecular mechanisms of KLF12 activating lipid consumption and browning still remains unclear. This is one of the limitations of this study, and further research is needed.

In summary, our study identified lncRNA COL18A1-AS1 was downregulated in ccRCC and could be a diagnostic and prognostic biomarker. DNA methylation at the CpG islands within its promoter of COL18A1-AS1 leaded to its low expression. Restoring the epigenetically silenced COL18A1-AS1 repressed tumor progression and promoted lipid browning and consumption in vitro and in vivo. Mechanistically, COL18A1-AS1 could competitively bind miR-1286 to increase the expression of KLF12. Therefore, we suggested COL18A1-AS1 might be a potential therapeutic target for ccRCC.

## Materials and methods

### Human ccRCC tissue samples and cell culture

Fifty pairs of matched ccRCC and adjacent normal renal tissues were collected from Department of Urology, Wuhan Union Hospital, Huazhong University of Science and Technology (HUST). Written informed consent was obtained from each patient before operation and this study was approved by the Ethic Committee of Human Research of HUST.

Human RCC cell lines (ACHN, A498, CAKI-1, OS-RC-2 and 786-O) and the control cell line HK-2 were purchased from the American Type Culture Collection in the USA (ATCC, Manassas, VA, USA). DMEM high-glucose (BI, Beit-Haemek, Israel) containing 10% fetal bovine serum (FBS, BI, Beit-Haemek, Israel) and 1% streptomycin–penicillin (Google Biotechnology, Wuhan, China) was applied for cell culture. And cells were incubated in a humidified atmosphere with 5% CO2 at 37 °C.

### Transient transfection and lentivirus infection assay

The si-RNA and plasmid vector sequences used for transfection in this study are shown in Supplementary Table 2. For cell transient transfection, Lipofectamine 3000 reagent (Invitrogen, Carlsbad, CA, USA) was used according to the manufacturer’s instructions when the RCC cell lines were at 50–60% confluence. For cell lentivirus infection, pLent-COL18A1-AS1-GFP-Puro lentivirus or empty vector pLent-GFP-Puro lentivirus (Vigene Biology) was applied when the RCC cell lines were at 40–50% confluence. T days later, 5 μg/ml puromycin was added to kill the cells that had not been transfected.

### RNA isolation and quantitative reverse transcription PCR (qRT-PCR)

The total RNA was extracted using Ultrapure RNA Kit according to the manufacturer’s instructions (CWBIO, Taizhou, China). The RNA was reverse transcribed into cDNA using a Superscript II reverse transcription kit (Vazyme, Nanjing, China). Then, we used a SYBR-Green master kit (Vazyme, Nanjing, China) on a quantitative real-time PCR instrument (Applied Biosystems, USA). GAPDH or U6 was used for standardization. The primers used to amplify all of mRNA and miRNA involved in this study were chemically synthesized by TSINGKE (TSINGKE, Beijing, China) and the primer sequences used for qRT-PCR are shown in Supplementary Table 3.

### RNA cellular fractionation assay

Cellular fraction assay of RNA was conducted with a PARIS™ Kit (Invitrogen, USA) according to the manufacturer’s instructions. GAPDH mRNA was a cytoplasmic control, U6 RNA was a nuclear control. Fractionated RNA was determined by qRT-PCR in triplicate.

### Fluorescence in situ hybridization (FISH)

Cy3-labeled COL18A1-AS1 probe, 18 S probe, and U6 probe were obtained from RiboBio (RiboBio, Guangzhou, China). 18 S probe was used as a positive control for the cytoplasm and U6 probe was for the nucleus. Cells in 24-well cell culture plate were fixed in 4% formaldehyde for 15 min at 50–60% confluence. Then, the Fluorescent in Situ Hybridization kit (RiboBio, Guangzhou, China) was used for the hybridization. Cells were visualized with fluorescence microscope (Olympus, Tokyo, Japan).

### Methylation-Specific PCR (MSP)

We isolated the genomic DNA from ccRCC and adjacent normal renal tissues with a TIANamp Genomic DNA Kit (TIANGEN BIOTECH, Beijing, China). Then, MSP primers were designed according to the genomic sequence of transcription state site of COL18A1-AS1 (Fig. 3F). The methylated (M) primers and unmethylated (U) primers used for monitoring bisulfate-induced changes were synthesized by TSINGKE (TSINGKE, Beijing, China).

### Bisulfite sequencing PCR (BSP)

After extracting genomic DNA as above, we designed the BSP primers according to the genomic sequence of transcription state site of COL18A1-AS1. Then, Bisulfite treatment of DNA, PCR amplification, Electrophoresis, Purify DNA from agarose gel, Transformation experiment using DH5α, PCR amplification identification, and DNA sequencing. Specific technical support was provided by Servicebio (Servicebio, Wuhan, China).

### The CRISPR/dCas9-mediated editing system

The CRISPR/dCas9-mediated editing system for COL18A1-AS1 specific demethylation contained four sgRNAs targeting promoter region of COL18A1-AS1, which achieved demethylation of COL18A1-AS1 promoter region through a series of multiple targets. This system was packaged in lentivirus in 293 T cells. Then, we conducted RCC cell lentivirus infection assays with above methods. The editing system was designed and purchased from Genechem (Genechem, Shanghai, China).

### Western blotting

Proteins from cells and tissues were extracted with RIPA lysis buffer (Servicebio, Wuhan, China) containing PMSF. Then, we subjected the protein to SDS-PACE and transferred to a polyvinylidene difluoride (PVDF) membrane (Millipore, Bedford, MA, USA). After that, the membrane was blocked in 5% skim milk for 2 hours at room temperature. Subsequently, the membranes were incubated with specific primary antibodies at 4°C over night. The next day, the membranes were washed with PBST for 3 times and incubated with the appropriate secondary antibodies for 2 h at room temperature. At last, the protein bands were visualized with Pierce™ ECL Western Blotting Substrate (Thermo Fisher Scientific, Waltham, MA, USA) using ChemiDoc XRS + (Bio‑Rad Laboratories, Inc.,). The detailed information on the antibodies was showed in Supplementary Table 4.

### Immunohistochemistry (IHC)

Tissue samples were fixed in formalin at room temperature for 12 h, dehydrated and embedded in paraffin. Then, EDTA was used to deparaffinize and rehydrated. After that, sections were incubated for 5 min at 120 °C for antigen retrieval. FBS was applied for blocking. Next, we incubated the sections over night at 4 °C with specific primary antibody. After incubating with the appropriate secondary antibodies for 2 h, slides were observed in three randomly selected fields under a light microscope (Olympus, Tokyo, Japan).

### Immunofluorescence (IF)

Cells were fixed in 4% formaldehyde at 50–60% confluence and blocked in 5% bovine serum albumin (BSA, Servicebio, Wuhan, China) for 30 min. Then, cells were incubated with specific primary antibody at 4 °C overnight, followed by incubation with the secondary antibody conjugated with FITC (abcam, Cambridge, UK). 4',6-diamidino-2-phenylindole (DAPI, Thermo Fisher Scientific, Waltham, MA, USA) was used for cell nuclei counterstaining. Lipids were stained with BODIPY (MedChemExpress, NJ, USA). Cells were visualized with fluorescence microscope (Olympus, Tokyo, Japan).

### RNA-binding protein immunoprecipitation (RIP)

We performed the RIP assays with a RIP™ RNA-binding protein immunoprecipitation kit (Millipore, Bedford, MA, USA) according to the manufacturer’s instructions. A total of 2 × 10^7^ cells were lysed with the RIP lysis buffer. Then, anti-AGO2 antibody or negative control anti-IgG antibody was incubated with magnetic beads for 30 min. Subsequently, we mixed the magnetic beads with cell lysate and incubated overnight at 4°C. Finally, we isolated the AGO2-binding RNA for the following qRT-PCR.

### Luciferase reporter assays

The luciferase vectors used in this study were constructed by Genechem (Genechem, Shanghai, China). The sequence of the COL18A1-AS1 containing the wild-type binding sequence or mutant binding sequence was cloned in a GV238-based vector. And the sequence of the KLF12 mRNA 3' UTR with wild-type binding sequence or mutant binding sequence was cloned in a GV238-based vector as well. CAKI-1 and 786-O cells were transfected with this vector. After that, the luciferase activity was measured with a Dual-Luciferase Reporter Assay Kit (Promega, Madison, WI, USA) from the cell lysate.

### Cell function experiments

Detailed descriptions of cell function experiments were listed in the Supplementary Materials and Methods. The experiments were performed in triplicate.

### Xenograft tumor and in vivo metastasis models

All animal experiments were approved by the Animal Ethics Committee of HUST. OS-RC-2 cells were stably transfected with COL18A1-AS1 overexpression or negative control lentivirus. A total of 2 × 10^6^ OS-RC-2 cells was subcutaneously injected into the upper back of four-week-old male BALB/c nude mice (HFK Biotechnology, Beijing, China) to construct the xenograft model (*n* = 5 mice/group). Tumor sizes were measured every week. Mice were sacrificed after 30 days. Tumor size and weight were finally measured and IHC assays were conducted.

To evaluate the metastasis ability of OS-RC-2 cells, we injected 1 × 10^6^ OS-RC-2 cells via tail vein of four-week-old male BALB/c nude mice (HFK Biotechnology, Beijing, China) to construct the metastasis model (*n* = 5 mice/group). 45 days later, nude mice were sacrificed and H&E staining were used to detect the lung and liver metastasis of tumor cells.

### Bioinformatics analysis

COL18A1-AS1 expression level and KLF12 expression level in ccRCC tissues together with clinical data were downloaded from The Cancer Genome Atlas database (TCGA, https://xenabrowser.net/heatmap/). The CpG island methylation analysis of COL18A1-AS1 was performed with MethPrimer (http://www.urogene.org/cgi-bin/methprimer/methprimer.cgi). Cancer Cell Line Encyclopedia (CCLE, https://docs.sevenbridges.com/docs/ccle) database was used to evaluate the methylation status of COL18A1-AS1 promoter in RCC cell lines. DIANA (http://diana.imis.athena-innovation.gr/DianaTools/), miRDB (http://mirdb.org/) and TARGETSCAN database (http://www.targetscan.org/vert_71/) were applied to predict the binding sequence of miR-1286, COL18A1-AS1 and KLF12. The gene set enrichment analysis (GSEA; http://software.broadinstitute.org/gsea/index.jsp) was conducted to explore the COL18A1-AS1 and KLF12 related signaling pathways.

### Statistical analysis

All statistics analysis in the present study were performed by GraphPad Prism 7.0 (GraphPad Software, San Diego, California, USA) and SPSS 22.0 (IBM, NY, USA). Normality test, Student’s *t*-test or paired Student’s *t*-test, receiver operator characteristic curve, Pearson *χ*2-test, univariate and multivariate Cox regression analysis, linear regression, Pearson correlation coefficient and Kaplan–Meier curve with log-rank test were conducted as indicated. The value was determined to be significance when *P* < 0.05.

## Supplementary information

Supplementary Methods

Supplementary Figure Legends

Supplementary Fig. 1

Supplementary Fig. 2

Supplementary Fig. 3

Supplementary Fig. 4

Supplementary Fig. 5

Supplementary Fig. 6

Supplementary Fig. 7

Supplementary Fig. 8

Supplementary Fig. 9

Supplementary Fig. 10

Supplementary Tables

Original Data File

Academic Journals Reporting Checklist

## Data Availability

All data generated or analyzed in this study are included in this paper and can be obtained from the corresponding author according to formal requirement.
