# Supplementary material for: Restoring the epigenetically silenced lncRNA COL18A1-AS1 represses ccRCC progression by lipid browning via miR-1286/KLF12 axis
Source: Cell Death Dis. 2022 Jul 4;13(7):578. doi: 10.1038/s41419-022-04996-2 (PMC9253045; doi:10.1038/s41419-022-04996-2)
Supplement: Supplementary file 1 — Supplementary Methods [file 41419_2022_4996_MOESM1_ESM.doc]

**SUPPLEMENTARY MATERIALS AND METHODS**

**Cell proliferation assays**

The Cell Counting Kit-8 (Dojindo, Kyushu Island, Japan) was used to measure the cell proliferation ability. RCC cells including ACHN, CAKI-1, OS-RC-2 and 786-O transfected with si-RNA or plasmids were grown in 96-well cell culture plate. The number of cells in each well was 1 x 10^3^ in with 100 μl of medium. Every 24 hours, CCK8 solution (10 ul) was added to each well. The absorbance of each well was measured at 450 nm after incubation of 3 h at 37℃.

For colony formation assay, 1 x 10^3^ cells were seeded into 6-well cell culture plate. About 2 weeks later, the cells were fixed and stained by crystal violet. After that, we observed the size and amount of the colonies.

**Cell migration and invasion assays**

For Transwell assays, a total of 1 x 10^4^ per well RCC cells which had been starved for 6-8 hours was seeded in the upper chamber with 200 μl of serum-free medium to measure the migration ability. Meanwhile, 2 x 10^4^ per well cells were added into the Matrigel‑coated upper transwell chambers for invasion ability. The bottom chamber was filled with 1 ml DMEM containing 10% FBS. 24 hours later, the migrated or invaded cells were fixed and stained with 0.1% crystal violet dye for 20 min. After that, we washed the chambers with PBS and cells were observed and counted in five randomly selected fields under a light microscope (Olympus, Tokyo, Japan).

For wound healing assays, we seeded RCC cells in 6-well cell culture plate. Then, we scraped the cell layer to make a scratch when cells at 80-90% confluence. Cells were cultured in DMEM containing 1% FBS at 37℃ for 24 hours. And the wound gaps were observed at 0 h, 12 h and 24 h respectively under a light microscope (Olympus, Tokyo, Japan) to evaluate the cell migration ability.

**Oil red staining**

RCC cells were seeded in 12-well plate, and were fixed with 4% formalin at the confluence of 50%. After washed with PBS for 3 times, cells were stained with oil red for 30 min at room temperature. Then, cells were observed under a light microscope (Olympus, Tokyo, Japan) after removed the excess oil red with PBS.

**Triglyceride detection**

About 5 x 10^5^ RCC cells were collected and 2% Triton X-100 (Solarbio, Beijing, China) was used to lyse cells. Then, the triglyceride assay kit (Jiancheng, Nanjing, China) was used to measure the triglyceride level of RCC cells according to the manufacturer’s protocols. 2.5 ul of cell lysate was added to 250 ul working solution and incubated at 37℃ for 10 min. Finally, the absorbance of each sample was measured at 510 nm with a spectrophotometer (Bio-rad, Hercules, CA, USA).
