# Supplementary material for: Restoring the epigenetically silenced lncRNA COL18A1-AS1 represses ccRCC progression by lipid browning via miR-1286/KLF12 axis
Source: Cell Death Dis. 2022 Jul 4;13(7):578. doi: 10.1038/s41419-022-04996-2 (PMC9253045; doi:10.1038/s41419-022-04996-2)
Supplement: Supplementary file 2 — Supplementary Figure Legends [file 41419_2022_4996_MOESM2_ESM.doc]

**Supplementary Fig. 1 RNA-Seq results of three pairs of ccRCC and adjacent normal tissues.** **A** COL18A1-AS1 was downregulated showed in the volcano plot. Red dot: upregulated genes. Blue dot: downregulated genes. Grey dot: unchanged genes. **B** COL18A1-AS1 was downregulated showed in the heatmap of top 20 genes with the lowest P value.

**Supplementary Fig. 2 Low COL18A1-AS1 expression was associated with poor OS in patients with ccRCC.** Patient samples from TCGA were separated into two groups: Those with low COL18A1-AS1 expression and those with high COL18A1-AS1 expression. OS sub-analysis in regards to COL18A1-AS1 expression were conducted in subgroups of patients with ccRCC: **A** Age ≤ 60 years old, **B** Age ＞ 60 years old, **C** Female, **D** Male, **E** G3 + G4, **F** T1 + T2, **G** Nx + N0, **H** Mx + M0, **I** M1 and **J** Stage I + Stage II.

**Supplementary Fig. 3 Low COL18A1-AS1 expression was associated with poor DFS in patients with ccRCC.** Patient samples from TCGA were separated into two groups: Those with low COL18A1-AS1 expression and those with high COL18A1-AS1 expression. DFS sub-analysis in regards to COL18A1-AS1 expression were conducted in subgroups of patients with ccRCC: **A** Age ≤ 60 years old, **B** Age ＞ 60 years old, **C** Male, **D** G1 + G2, **E** G3 + G4, **F** Nx + N0 and **G** Mx + M0.

**Supplementary Fig. 4 Signaling pathways related with COL18A1-AS1. A** GSEA for the correlations between the free fatty acid oxidation, tumorigenesis, proliferation and epithelial-mesenchymal transition signaling pathways in ccRCC with the expression levels of COL18A1-AS1, according to GEO database (GSE53757). FDR < 0.25 and P < 0.05 were considered statistically significant. **B** qRT-PCR assays were applied to analyze the mRNA expression level of COL18A1 after transfection by si-RNA or COL18A1-AS1 overexpression vector for 24 h in RCC cells. **C** Western blot assays were applied to analyze the protein expression level of COL18A1 after transfection by si-RNA or COL18A1-AS1 overexpression vector for 48 h in RCC cells. **D, E** Cell migration and invasion ability of RCC cells was measured with Transwell assays or wound healing assays (Magnification: ×100 for Transwell assays and x40 for wound healing assays). *P < 0.05, **P < 0.01, ***P < 0.001, ****P < 0.0001. Error bars indicate mean ± SD. All the experiments were performed in triplicate.

**Supplementary Fig. 5 DNA methylation was correlated with the expression level of COL18A1-AS1 in ccRCC. A** The expression of COL18A1-AS1 was negatively correlated with COL18A1-AS1 DNA methylation according to TCGA database. **B** Distribution of 8 COL18A1-AS1 DNA promoter CpG sites. Correlation between COL18A1-AS1 expression level and 8 CpG sites of COL18A1-AS1 DNA promoter, including: **C** cg11581903, **D** cg02417576, **E** cg24031597, **F** cg02706881, **G** cg07529658, **H** cg05349039, **I** cg05275605, **J** cg17611512. **K** CGs distribution of BSP. **L** CGs distribution of MSP. **M** Cancer Cell Line Encyclopedia (CCLE) database was used to evaluate the methylation status of the COL18A1-AS1 promoter in different RCC cell lines (ACHN, A498, CAKI-1, OS-RC-2 and 786-O). *P < 0.05, **P < 0.01, ***P < 0.001, ****P < 0.0001. Error bars indicate mean ± SD.

**Supplementary Fig. 6 The CRISPR/dCas9-mediated editing system for COL18A1-AS1 specific demethylation. A** Schematic diagram of the working principle of the CRISPR/dCas9-mediated editing system for specific demethylation. **B** Schematic diagram of CRISPR/dCas9-mediated editing system for COL18A1-AS1 specific demethylation. **C, D** Construction sequence of CRISPR/dCas9-mediated editing system for COL18A1-AS1 specific demethylation.

**Supplementary Fig. 7 COL18A1-AS1 interacted with miR-1286 in ccRCC. A, B** Cell proliferation ability of CAKI-1 cells co-transfected with COL18A1-AS1 vector and/or miR-1286 MIMICS was determined using CCK8 assays or colony formation assays. **C, D** Cell migration and invasion ability of CAKI-1 cells was measured with Transwell assays or wound healing assays (Magnification: ×100 for Transwell assays and x40 for wound healing assays). **E** Photomicrographs of Oil Red staining of CAKI-1 cells. Scale bars, 10 μm. **F** Relative TG (mmol/gprot) levels in CAKI-1 cells assessed by a triglyceride assay kit. **G** Representative FISH images showed the co-localization of COL18A1-AS1 (red) and miR-1286 (green) in the cytoplasm of ACHN cells. Cell nuclear appear in blue (DAPI). Scale bars, 10 μm. *P < 0.05, **P < 0.01, ***P < 0.001, ****P < 0.0001. Error bars indicate mean ± SD. All the experiments were performed in triplicate.

**Supplementary Fig. 8 Kaplan-Meier curves for OS analysis of 16 candidate genes. A** ALPK1, P = 0.0055. **B** C20orf197, P = 0.7791. **C** CHIA, P = 0.2414. **D** CHIC1, P < 0.0001. **E** DIRAS1, P = 0.0319. **F** DYNAP, P = 0.0023. **G** F8, P = 0.0022. **H** HIF1AN, P = 0.0040. **I** IPCEF1, P = 0.0817. **J** KLF12, P = 0.0002. **K** LETMD1, P = 0.3962. **L** MAFB, P = 0.8304. **M** PITPNM3, P = 0.2901. **N** STK16, P = 0.9290. **O** SYT4, P = 0.8029. **P** TSPAN11, P = 0.7782.

**Supplementary Fig. 9 COL18A1-AS1/miR-1286/KLF12 axis repressed ccRCC progression. A** Western blot analysis of KLF12 protein in ACHN and 786-O cells co-transfected with si-COL18A1-AS1 and/or miR-1286 inhibitor. **B** Representative FISH and IF images showed the expression level of COL18A1-AS1 (red) and KLF12 (green) in ACHN cells. Cell nuclear appear in blue (DAPI). Scale bars, 10 μm. **C, D** Cell proliferation ability of CAKI-1 cells co-transfected with COL18A1-AS1 vector and/or si-KLF12 was determined using CCK8 assays or colony formation assays. **E, F** Cell migration and invasion ability of CAKI-1 cells was measured with Transwell assays or wound healing assays (Magnification: ×100 for Transwell assays and x40 for wound healing assays). **G** Photomicrographs of Oil Red staining of CAKI-1 cells. Scale bars, 10 μm. **H** Relative TG (mmol/gprot) levels in CAKI-1 cells assessed by a triglyceride assay kit. *P < 0.05, **P < 0.01, ***P < 0.001, ****P < 0.0001. Error bars indicate mean ± SD. All the experiments were performed in triplicate.

**Supplementary Fig. 10 KLF12 positively regulated marker genes of lipid browning. A** mRNA levels of lipid browning marker genes (UCP1, PGC1A, CIDEA and DIO2) in ACHN and 786-O cells transfected with si-KLF12 assessed by qRT-PCR. *P < 0.05, **P < 0.01, ***P < 0.001, ****P < 0.0001. Error bars indicate mean ± SD. All the experiments were performed in triplicate.
