# Supplementary figures and images for: Restoring the epigenetically silenced lncRNA COL18A1-AS1 represses ccRCC progression by lipid browning via miR-1286/KLF12 axis
Source: Cell Death Dis. 2022 Jul 4;13(7):578. doi: 10.1038/s41419-022-04996-2 (PMC9253045; doi:10.1038/s41419-022-04996-2)

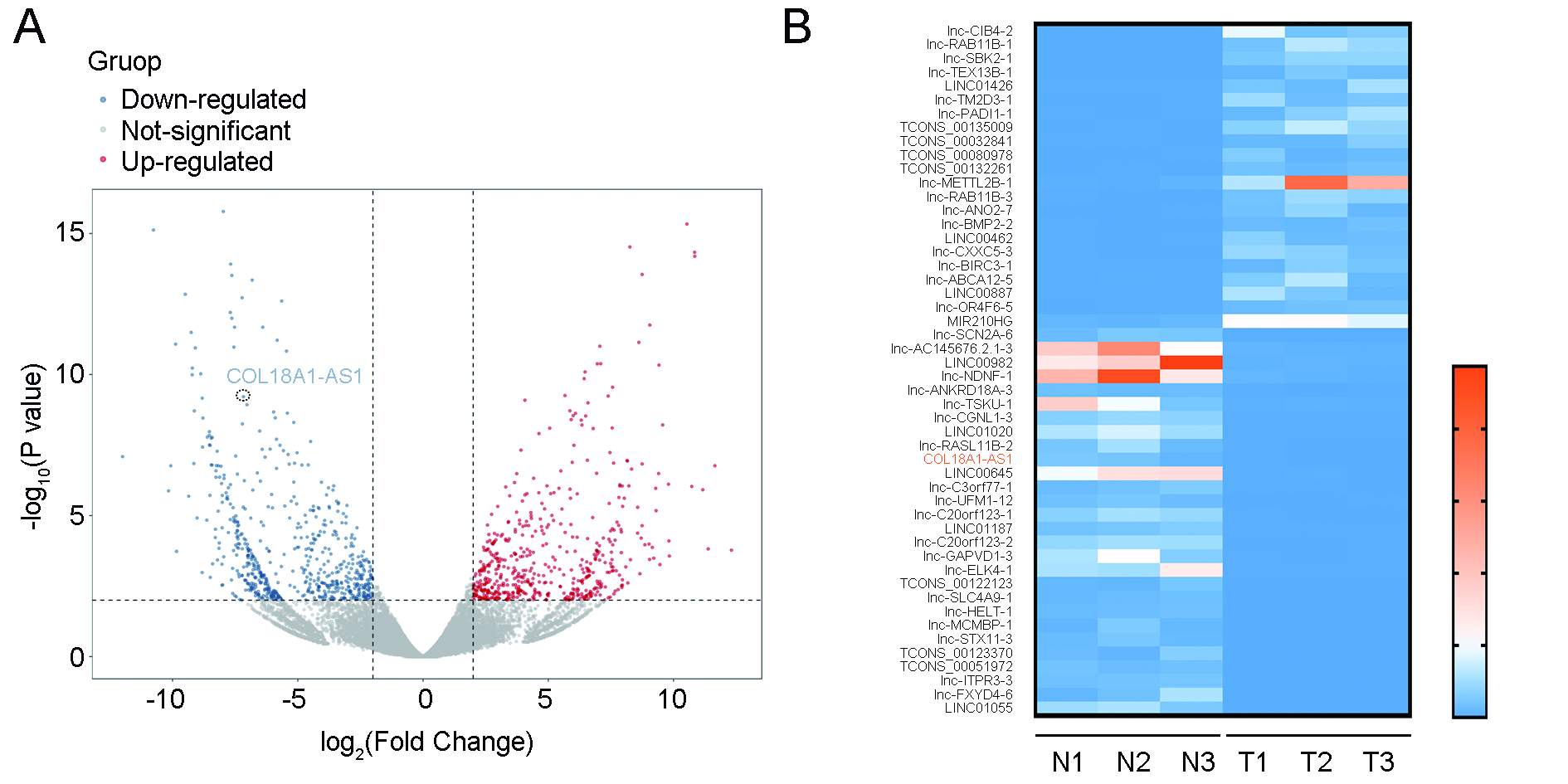

Supplement: Supplementary file 3 — Supplementary Fig. 1 [file 41419_2022_4996_MOESM3_ESM.tif]

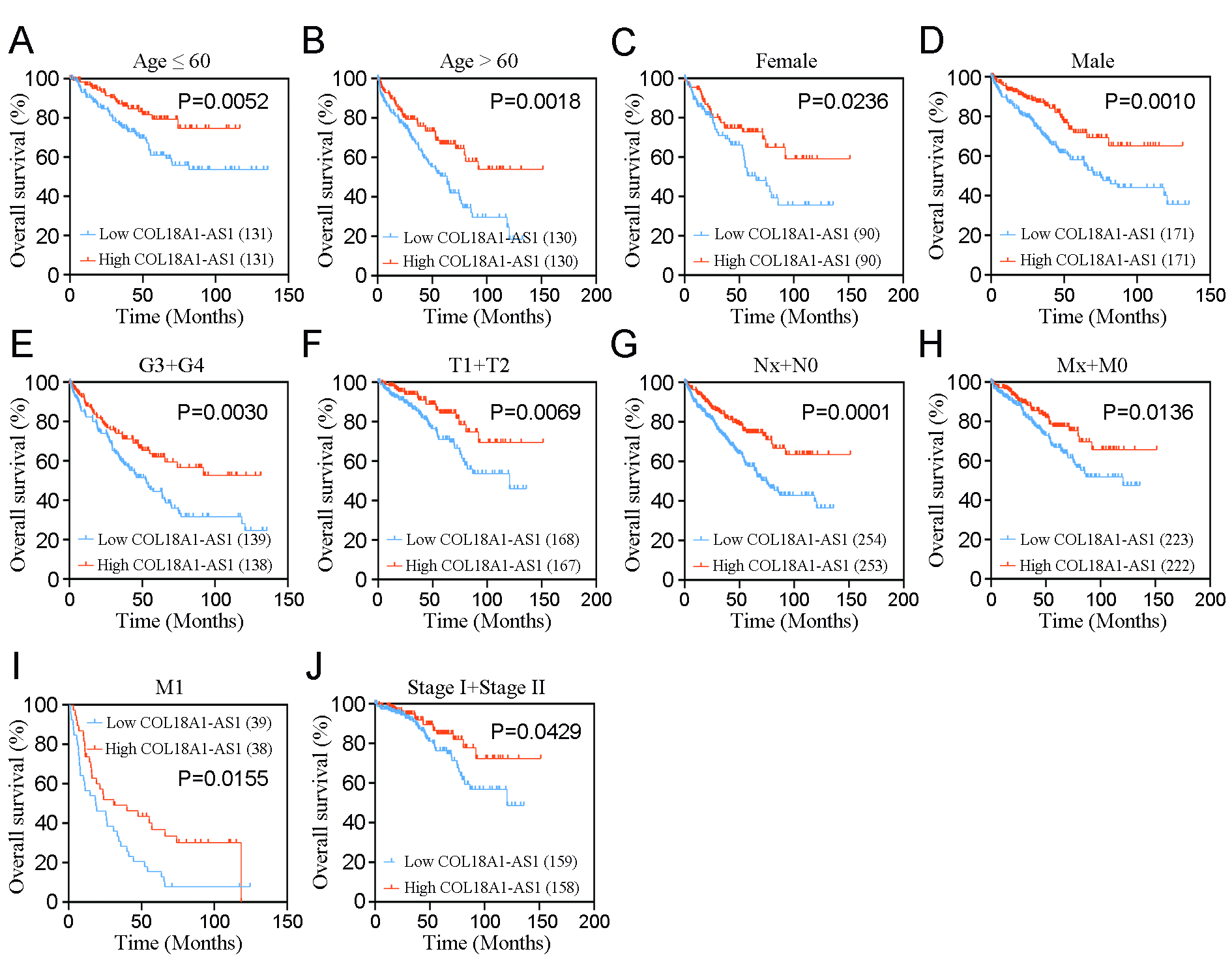

Supplement: Supplementary file 4 — Supplementary Fig. 2 [file 41419_2022_4996_MOESM4_ESM.tif]

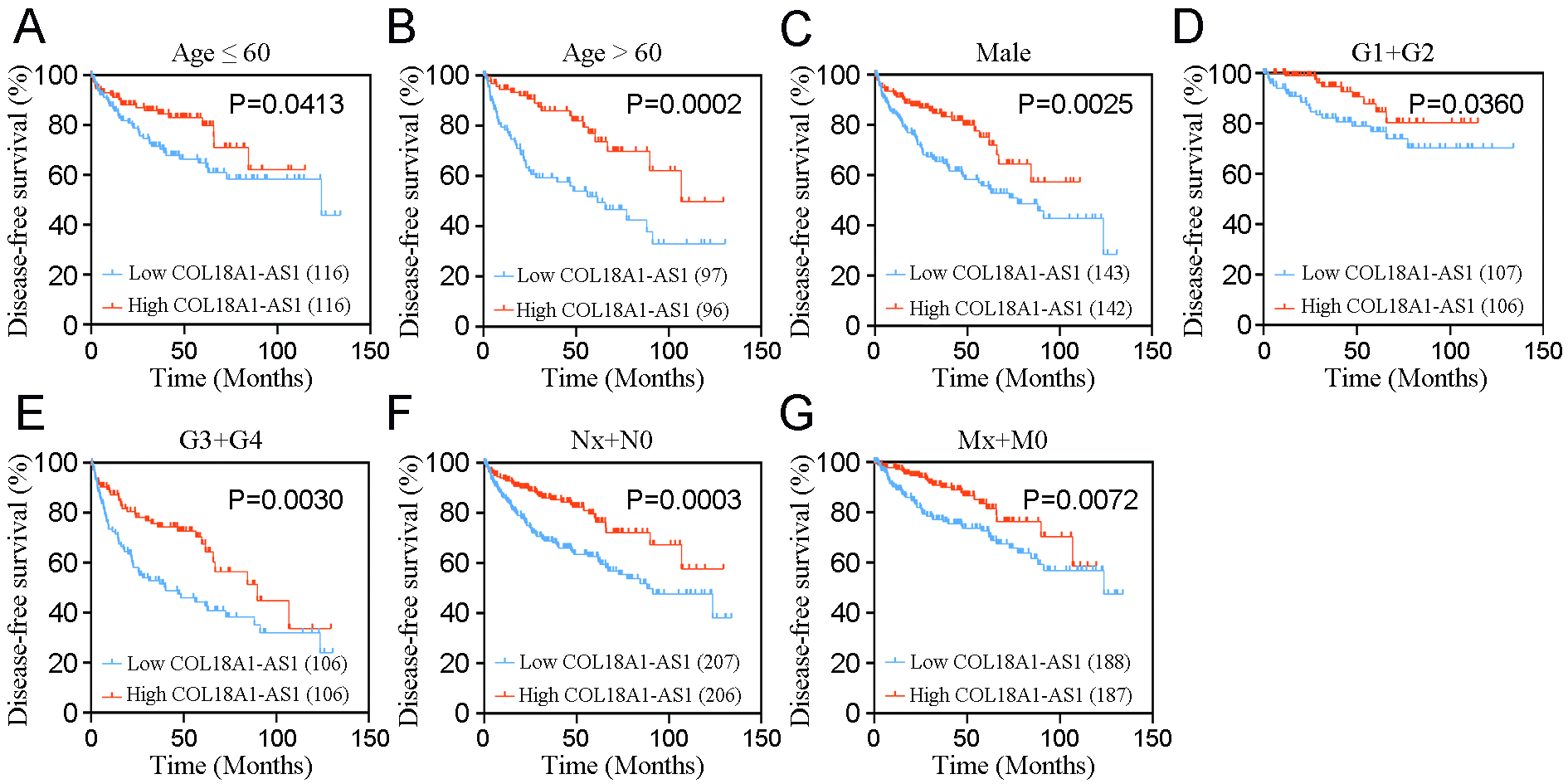

Supplement: Supplementary file 5 — Supplementary Fig. 3 [file 41419_2022_4996_MOESM5_ESM.tif]

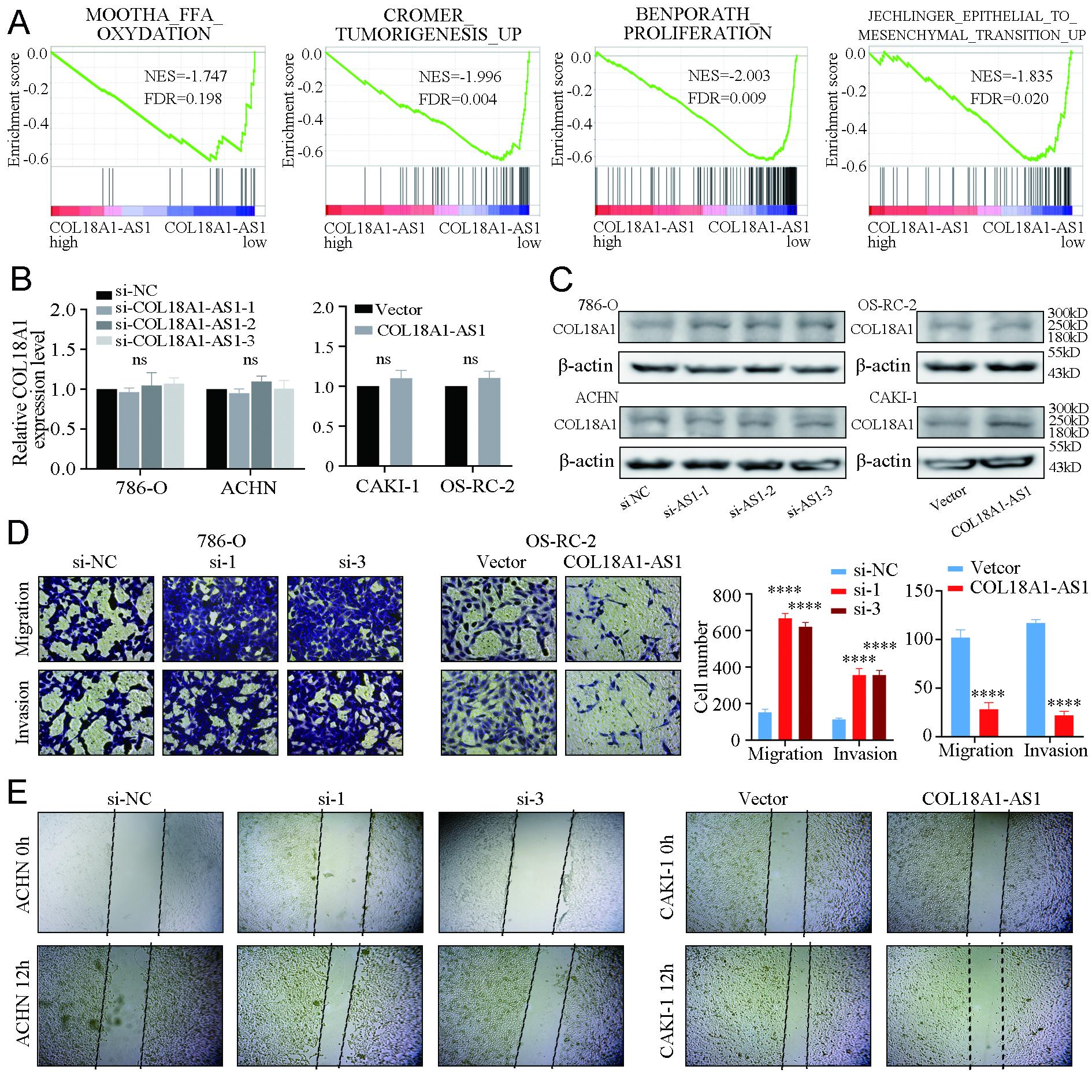

Supplement: Supplementary file 6 — Supplementary Fig. 4 [file 41419_2022_4996_MOESM6_ESM.tif]

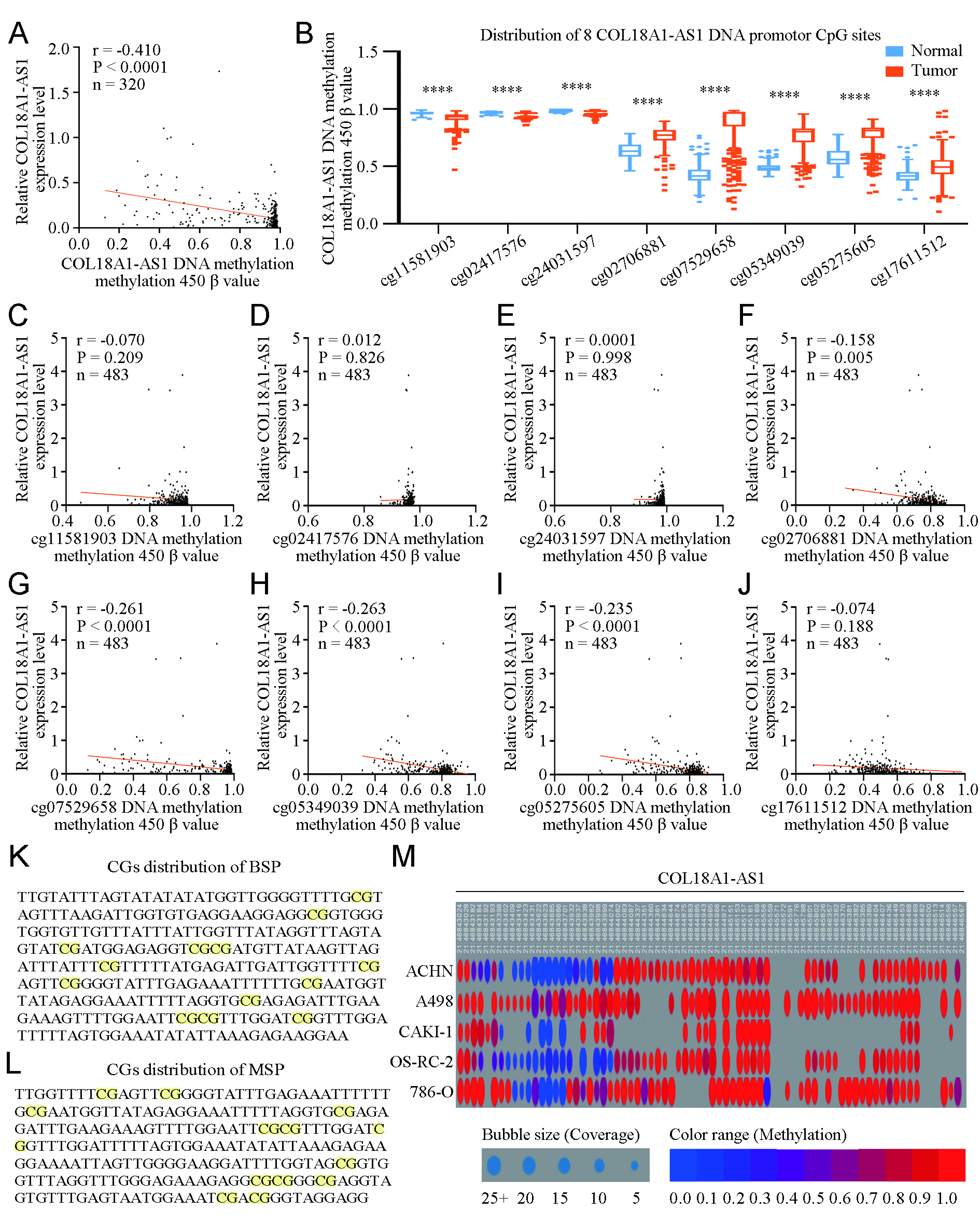

Supplement: Supplementary file 7 — Supplementary Fig. 5 [file 41419_2022_4996_MOESM7_ESM.tif]

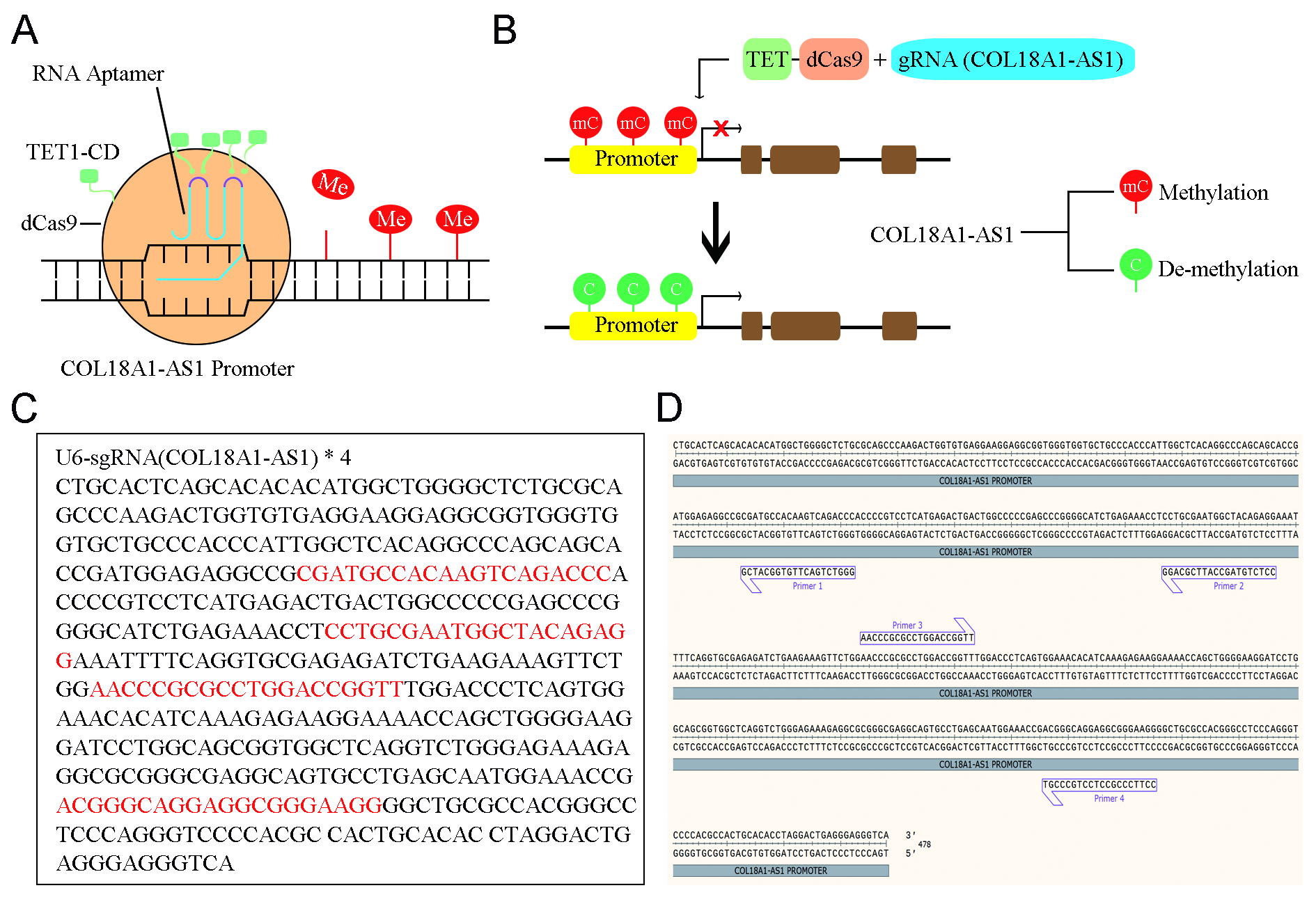

Supplement: Supplementary file 8 — Supplementary Fig. 6 [file 41419_2022_4996_MOESM8_ESM.tif]

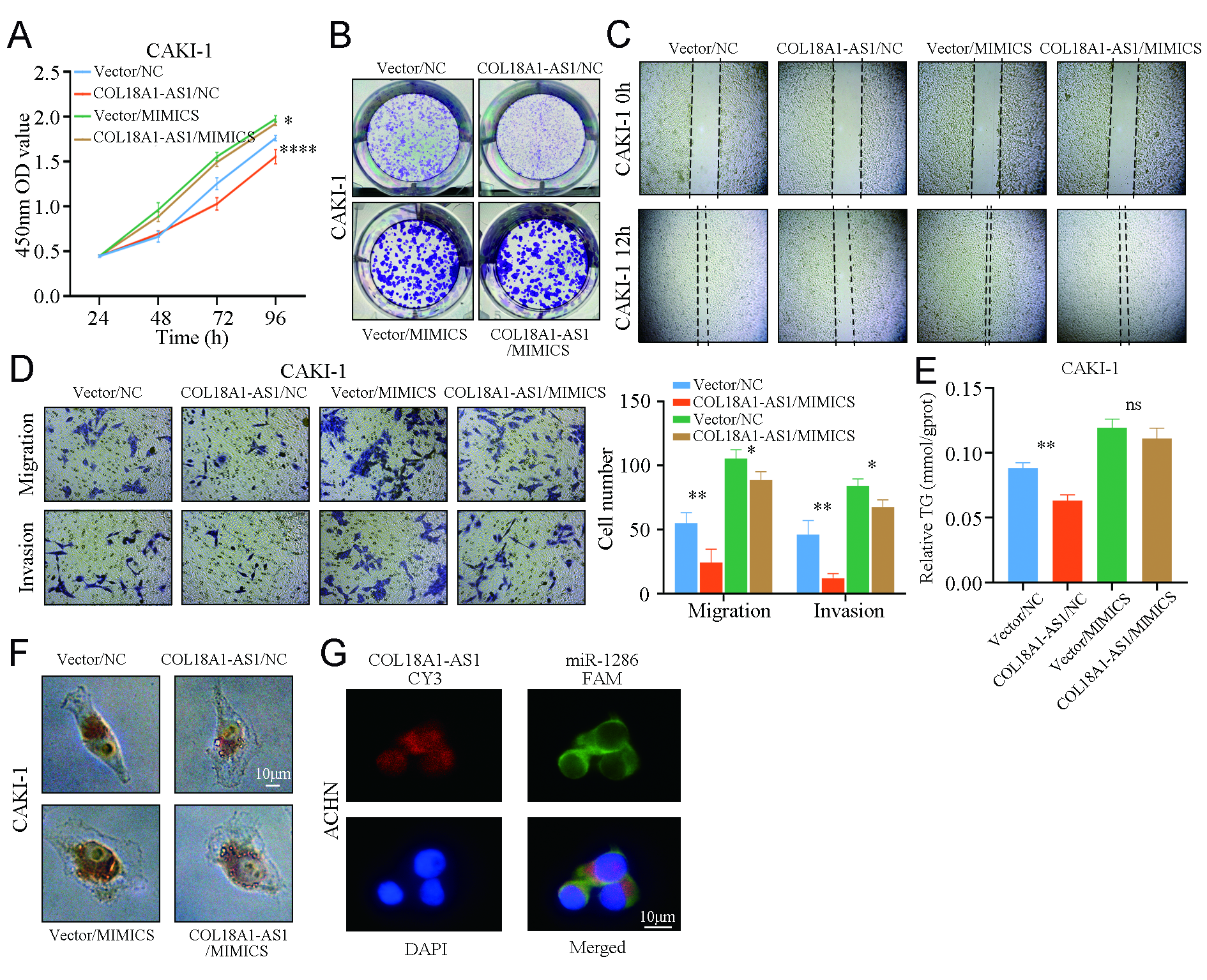

Supplement: Supplementary file 9 — Supplementary Fig. 7 [file 41419_2022_4996_MOESM9_ESM.tif]

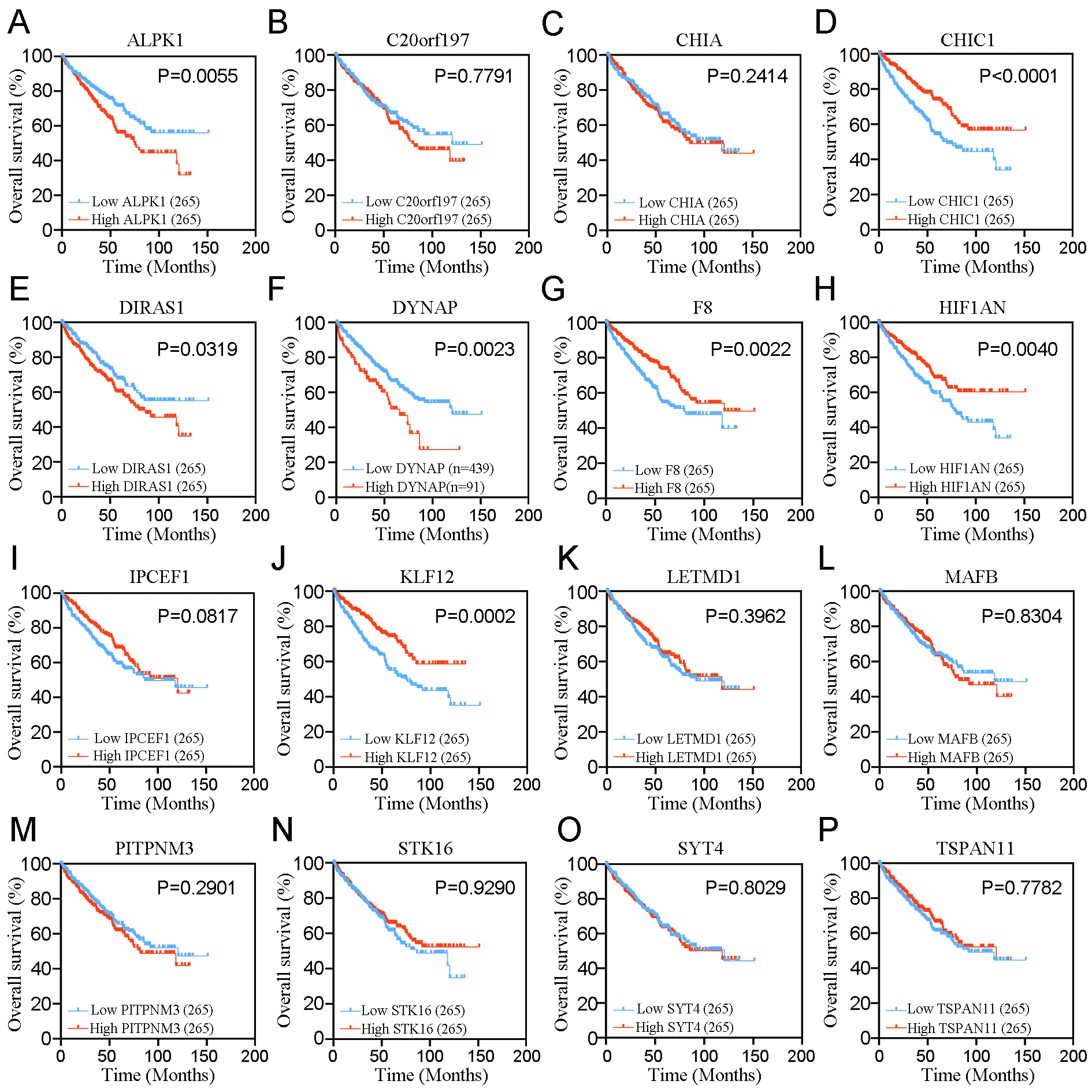

Supplement: Supplementary file 10 — Supplementary Fig. 8 [file 41419_2022_4996_MOESM10_ESM.tif]

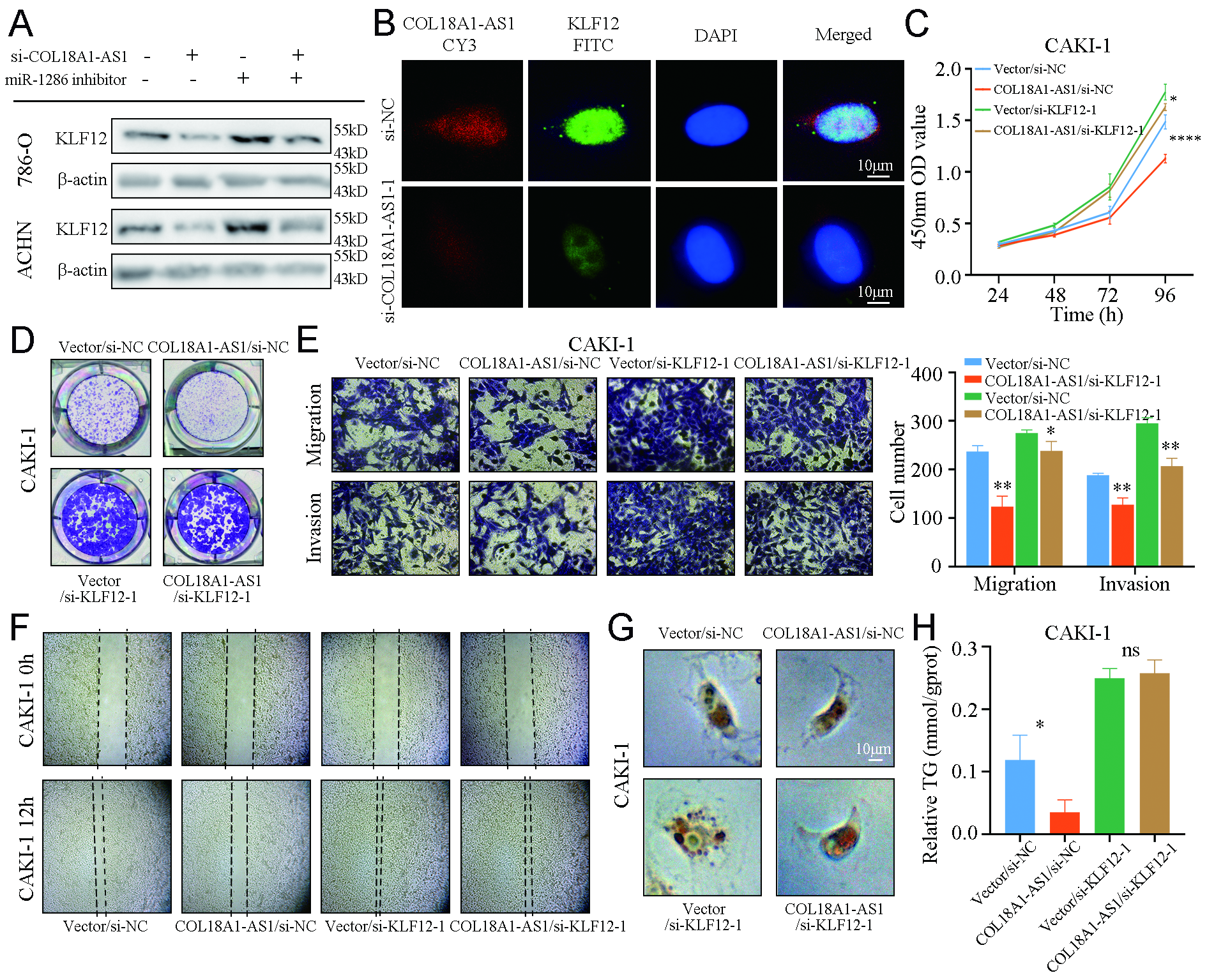

Supplement: Supplementary file 11 — Supplementary Fig. 9 [file 41419_2022_4996_MOESM11_ESM.tif]

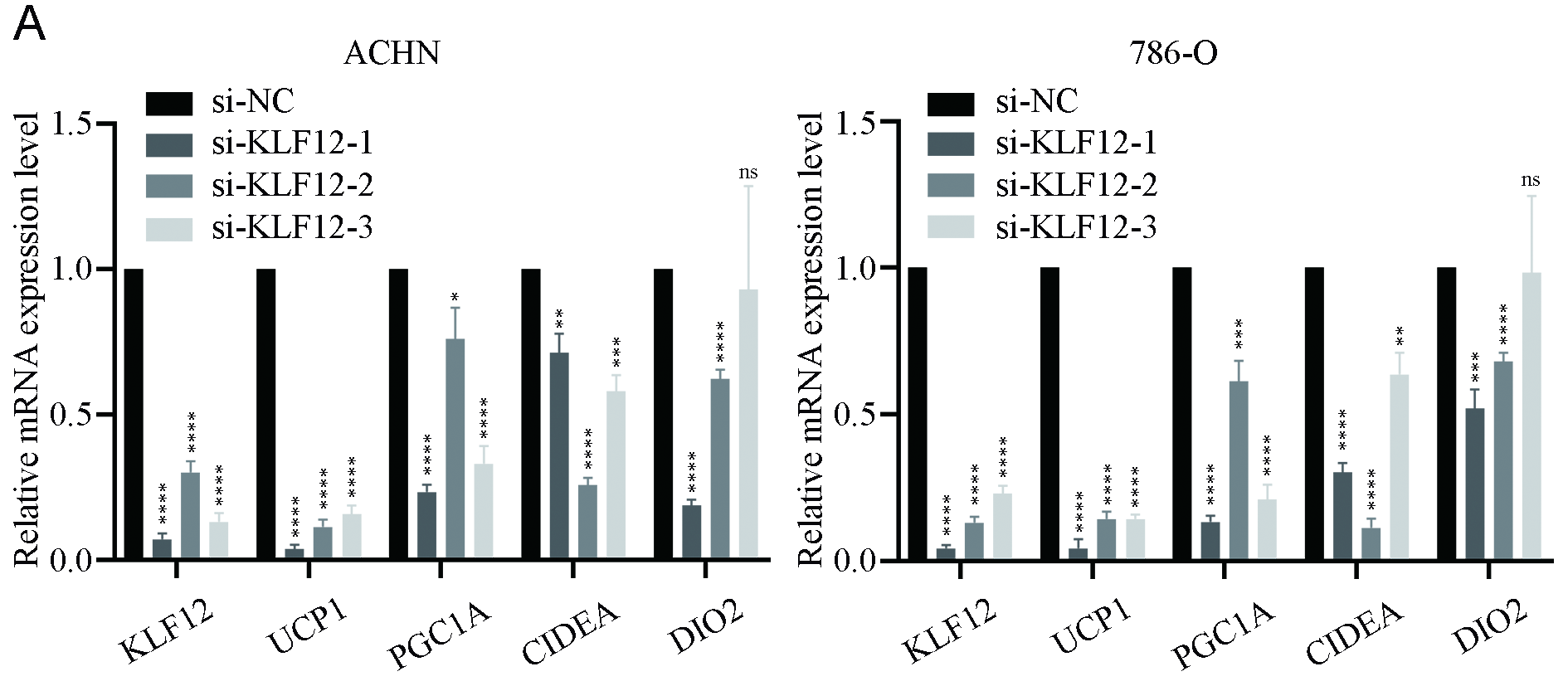

Supplement: Supplementary file 12 — Supplementary Fig. 10 [file 41419_2022_4996_MOESM12_ESM.tif]
