# Supplementary material for: Restoring the epigenetically silenced lncRNA COL18A1-AS1 represses ccRCC progression by lipid browning via miR-1286/KLF12 axis
Source: Cell Death Dis. 2022 Jul 4;13(7):578. doi: 10.1038/s41419-022-04996-2 (PMC9253045; doi:10.1038/s41419-022-04996-2)
Supplement: Supplementary file 13 — Supplementary Tables [file 41419_2022_4996_MOESM13_ESM.docx]

**Supplementary Table 1.** Univariate and multivariate analyses of COL18A1-AS1 level and patient overall survival.

| Variables |  | Univariate analysis |  |  |  | Multivariate analysis^c^ |  |  |
| --- | --- | --- | --- | --- | --- | --- | --- | --- |
|  | HR^a^ | 95% CI^b^ | P value |  | HR | 95% CI | P value |  |
| Overall survival (n=522) |  |  |  |  |  |  |  |  |
| COL18A1-AS1  Low (n=261)  High (n=261) | 0.533 | 0.388-0.734 | <0.001 |  | 0.719 | 0.517-0.999 | 0.049 |  |
| Age (years)  <60 (n=244)  ≥60 (n=278) | 1.786 | 1.302-2.450 | <0.001 |  | 1.561 | 1.130-2.155 | 0.007 |  |
| Gender  Female (n=180)  Male (n=342) | 0.933 | 0.683-1.275 | 0.663 |  |  |  |  |  |
| T stage  T1 or T2 (n=335)  T3 or T4 (n=187) | 3.209 | 2.361-4.364 | <0.001 |  | 1.672 | 1.103-2.319 | 0.013 |  |
| N stage  N0 or NX (n=507)  N1 (n=15) | 3.944 | 2.135-7.285 | <0.001 |  | 2.651 | 1.414-4.969 | 0.002 |  |
| M stage  M0 or MX (n=445)  M1 (n=77) | 4.351 | 3.180-5.951 | <0.001 |  | 2.519 | 1.755-3.615 | <0.001 |  |
| G grade  G1 or G2 or GX (n=245)  G3 or G4 (n=277) | 2.715 | 1.925-3.827 | <0.001 |  | 1.716 | 1.186-2.485 | 0.004 |  |

a Hazard ratio, estimated from Cox proportional hazard regression model.

b Confidence interval of the estimated HR.

c Multivariate models were adjusted for T, N, M classification, age and gender.

**Supplementary Table 2.** siRNA and plasmid vector sequence used in this study.

| Name | Sequence (5’ - 3’) | Company |
| --- | --- | --- |
| si-COL18A1-AS1-1 | TTAACAGCATGACAAGCCA | RiboBio |
| si-COL18A1-AS1-2 | CCAAGGTTGTGAGAAGATT | RiboBio |
| si-COL18A1-AS1-3 | AGCTCTGAGATCACAGTGT | RiboBio |
| pcDNA3.1-COL18A1-AS1 | NR_027498.1 (2341bp) | VigeneBio |
| hsa-miR-1286 MIMICS | UGCAGGACCAAGAUGAGCCCU | QijingBio |
| hsa-miR-1286 inhibitor | AGGGCUCAUCUUGGUCCUGCA | QijingBio |
| si-KLF12-1 | GUGACCUUAGAUAGCGUUAAUTT | QijingBio |
| si-KLF12-2 | CCUUCAAGUCCCAUGAAUUTT | QijingBio |
| si-KLF12-3 | GCCAAAUGUGACCUUAGAUTT | QijingBio |

**Supplementary Table 3.** Primers sequences used in this study.

| Primers used for quantitative RT-PCR | | |
| --- | --- | --- |
| Name | Forward-primer (5’ - 3’) | Reverse-primer (5’ - 3’) |
| COL18A1-AS1 | AACCTCCTGCGAATGGCTAC | GTCGGTTGGTGAAAAACGGG |
| COL18A1 | TCTTCCGTGACTTCTCACTGC | CTGGAACTCCTCACAGTCCAC |
| ALPK1 | TGACCACCATTTGCTGTCC | ACGTGCCACGGATATTCAC |
| CHIC1 | ACCTCGGCAGGTTCAAACTC | GATGCTCCTCGCTCACTACC |
| DIRAS1 | GCCAGCGGACACCGGAGC | ACCACGCGGTAATCGTTACTCT |
| DYNAP | TAACTGGGGTCTGCGTGAAC | AGGAGAGGGTGTTCCAGGTT |
| F8 | TGCCCTGATGAGGTGCAAAG | CCATTCCCAATGGCATGCTG |
| HIF1AN | GTACTGGTGGCATCACATAGAG | CTGATGGGCTTTGAGAGGATATT |
| KLF12 | CGGCAGTCAGAGTCAAAACAG | CGGCTTCCATATCGGGATAGT |
| GAPDH | AAAAGCATCACCCGGAGGAGAA | AAGGAAATGAATGGGCAGCCG |
| U6 | TGCGGGTGCTCGCTTCGGCAGC | CCAGTGCAGGGTCCGAGGT |

| Primers used for miRNA | | |  |
| --- | --- | --- | --- |
| Name | Forward-primer (5’ - 3’) | Reverse-primer (5’ - 3’) | RT-primer (5’ - 3’) |
| miR-1286 | GGTGCAGGACCAAGATGAG | GCAGGGTCCGAGGTATTC | GTCGTATCCAGTGCAGGGTCCGAGGTATTCGCACTGGATACGACAGGGCT |
| miR-6887-3p | GCTCCCCTCCACTTTCC | GCAGGGTCCGAGGTATTC | GTCGTATCCAGTGCAGGGTCCGAGGTATTCGCACTGGATACGACCTAGGA |
| miR-6509-3p | GCGTTTCCACTGCCACTAC | GCAGGGTCCGAGGTATTC | GTCGTATCCAGTGCAGGGTCCGAGGTATTCGCACTGGATACGACAAATTA |
| miR-8073 | GGCAGCAGGGAGCG | CAGTGCGTGTCGTGGAGT | GTCGTATCCAGTGCGTGTCGTGGAGTCGGCAATTGCACTGGATACGACACGACG |
| miR-6132 | GAGCAGGGCTGGGGAT | CAGTGCGTGTCGTGGAGT | GTCGTATCCAGTGCGTGTCGTGGAGTCGGCAATTGCACTGGATACGACTGCAAT |
| miR-4667-3p | CGTTCCCTCCTTCTGTCC | GCAGGGTCCGAGGTATTC | GTCGTATCCAGTGCAGGGTCCGAGGTATTCGCACTGGATACGACCTGTGG |
| miR-6868-3P | CGGTTCCTTCTGTTGTCTGT | GCAGGGTCCGAGGTATTC | GTCGTATCCAGTGCAGGGTCCGAGGTATTCGCACTGGATACGACCTGCAC |
| miR-7157-5p | GTTCAGCATTCATTGGCAC | GCAGGGTCCGAGGTATTC | GTCGTATCCAGTGCAGGGTCCGAGGTATTCGCACTGGATACGACTCTCTG |
| miR-942-5p | GCGTCTTCTCTGTTTTGGC | CAGTGCGTGTCGTGGAGT | GTCGTATCCAGTGCGTGTCGTGGAGTCGGCAATTGCACTGGATACGACCACATG |
| miR-3116 | GCGTGCCTGGAACATAGTAG | CAGTGCGTGTCGTGGAGT | GTCGTATCCAGTGCGTGTCGTGGAGTCGGCAATTGCACTGGATACGACAGTCCC |
| U6 | Designed and synthesized by RiboBio. | | |

**Supplementary Table 4.** Antibodies used in this study.

| Name | Company | Catalog Number | Assay |
| --- | --- | --- | --- |
| COL18A1 | Abclonal | #A1722 | WB |
| KLF12 | Abclonal | #A20469 | WB, IF, IHC |
| AGO2 | Abcam | #ab186733 | RIP |
| UCP1 | Abclonal | #A5857 | WB |
| PGC1A | Abclonal | #A19674 | WB |
| CIDEA | Santa Cruz | #sc-293289 | WB |
| DIO2 | Proteintech | #26513-1-AP | WB |
| Ki67 | Proteintech | #27309-1-AP | IHC |
| β-actin | Proteintech | #60008-1-Ig | WB |
| HRP-conjugated Affinipure Goat Anti-Mouse IgG(H+L) | Proteintech | #SA00001-1 | WB |
| HRP-conjugated Affinipure Goat Anti-Rabbit IgG(H+L) | Proteintech | # SA00001-2 | WB |
