# Supplementary material for: Restoring the epigenetically silenced lncRNA COL18A1-AS1 represses ccRCC progression by lipid browning via miR-1286/KLF12 axis
Source: Cell Death Dis. 2022 Jul 4;13(7):578. doi: 10.1038/s41419-022-04996-2 (PMC9253045; doi:10.1038/s41419-022-04996-2)
Supplement: Supplementary file 14 — Original Data File [file 41419_2022_4996_MOESM14_ESM.docx]

**Fig. 5E**

**KLF12**


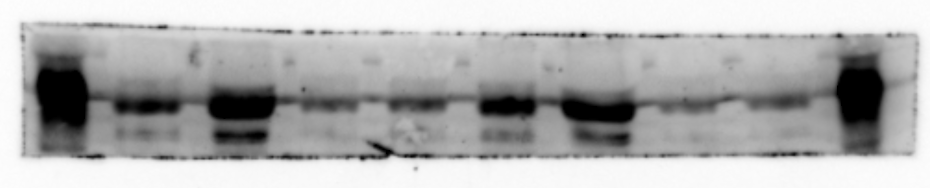


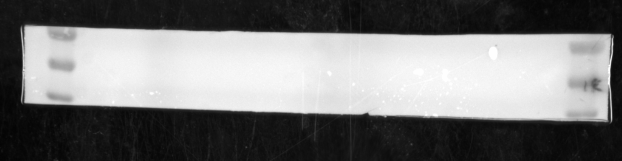


70kD

55kD

43kD

**β-actin**


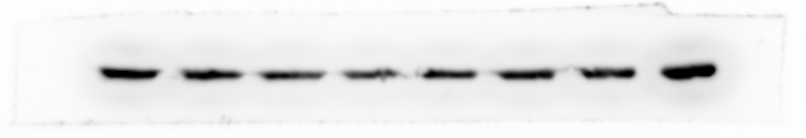


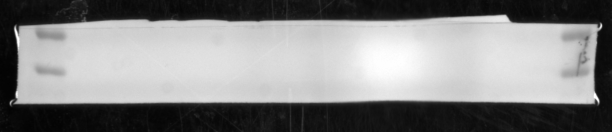


55kD

43kD

**Fig. 5H**

**KLF12**


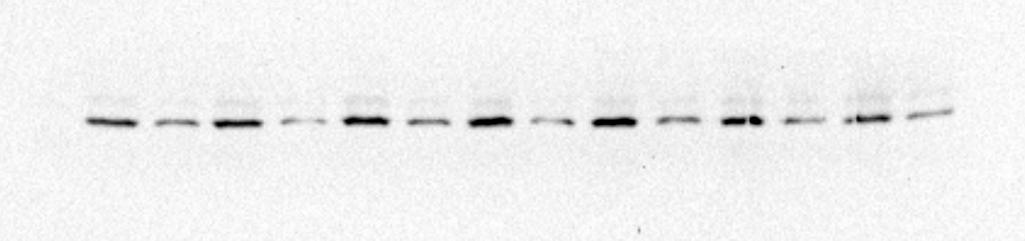


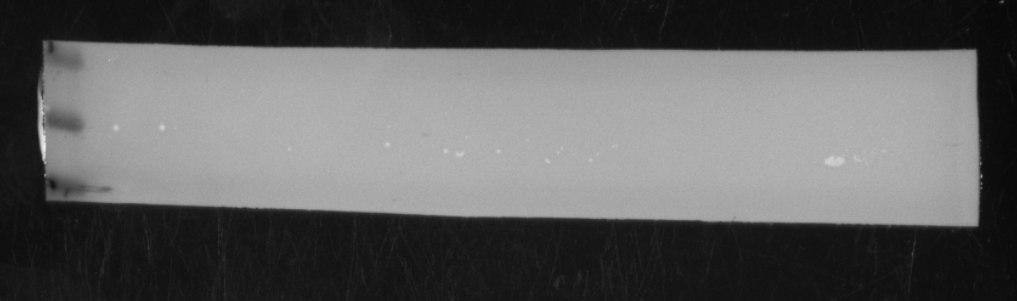


70kD

55kD

43kD

**β-actin**


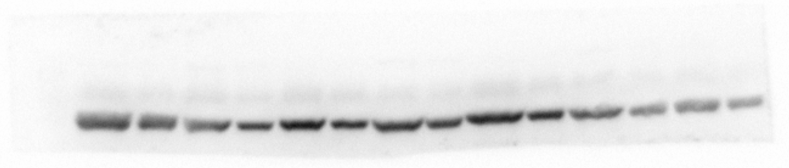


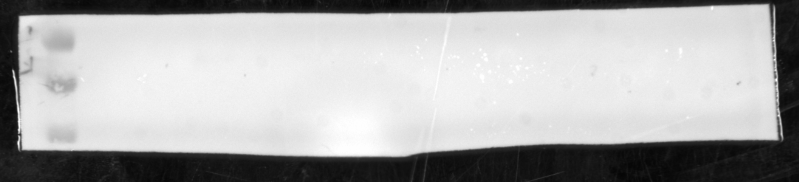


70kD

55kD

43kD

**KLF12**


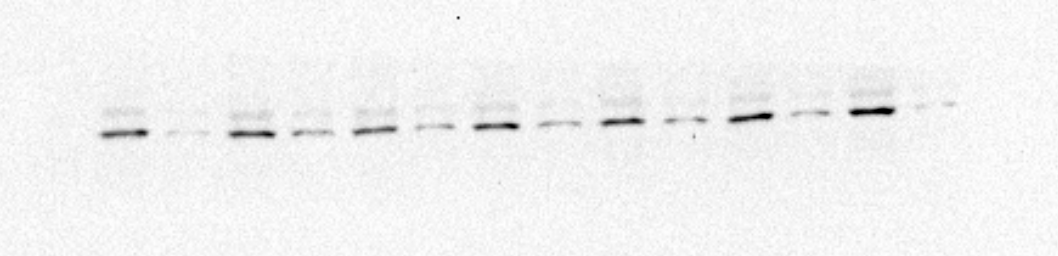


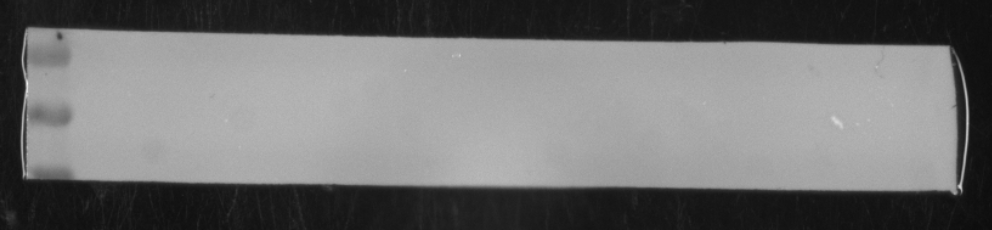


70kD

55kD

43kD

**β-actin**


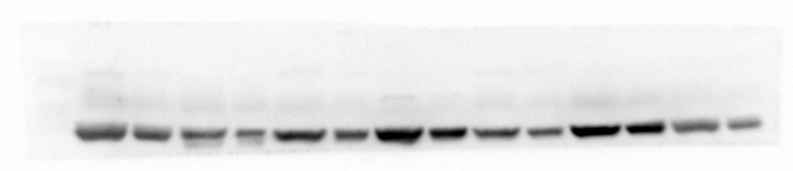


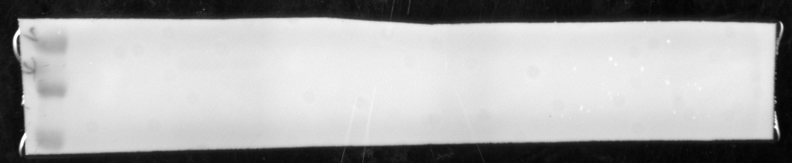


70kD

55kD

43kD

**Fig. 5K**

**KLF12**


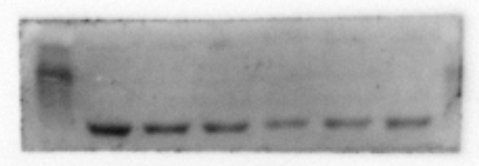


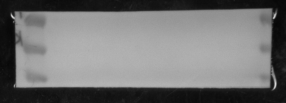


70kD

55kD

43kD

**β-actin**


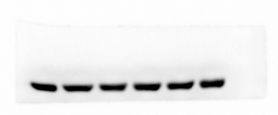


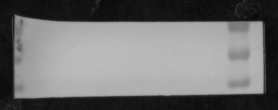


70kD

55kD

43kD

**Fig. 6B**

**KLF12**


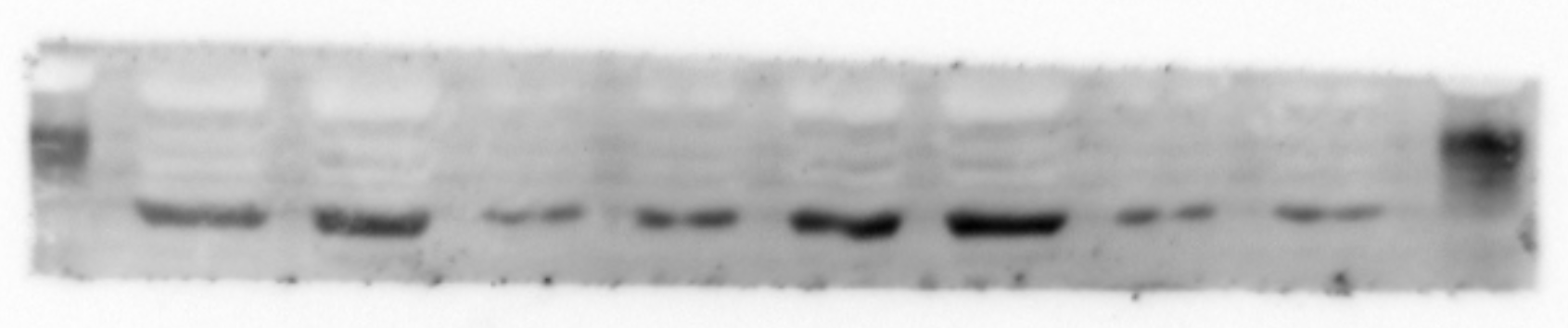


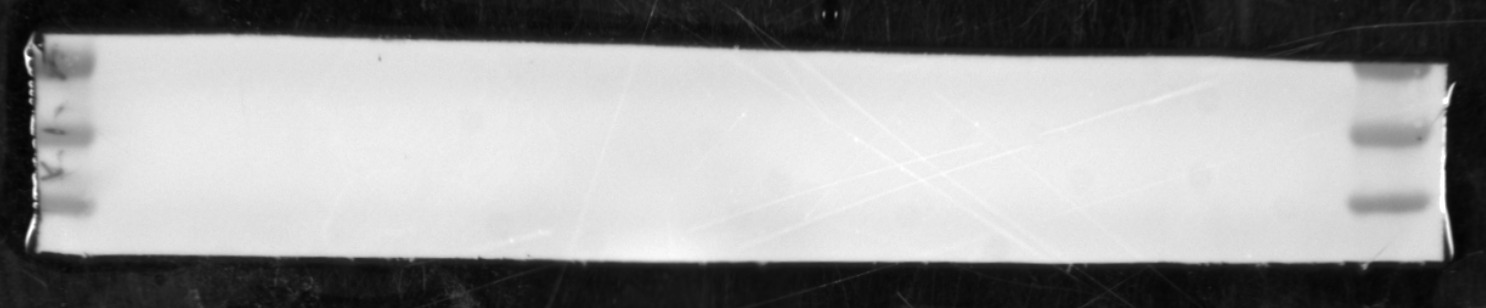


70kD

55kD

43kD

**β-actin**


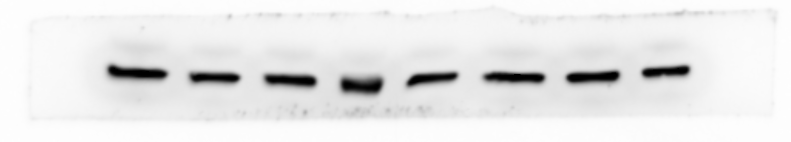


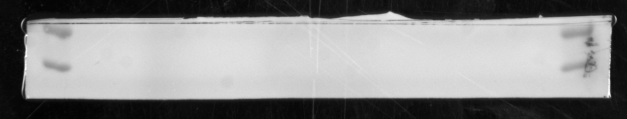


55kD

43kD

**Fig. 6J**

**KLF12**


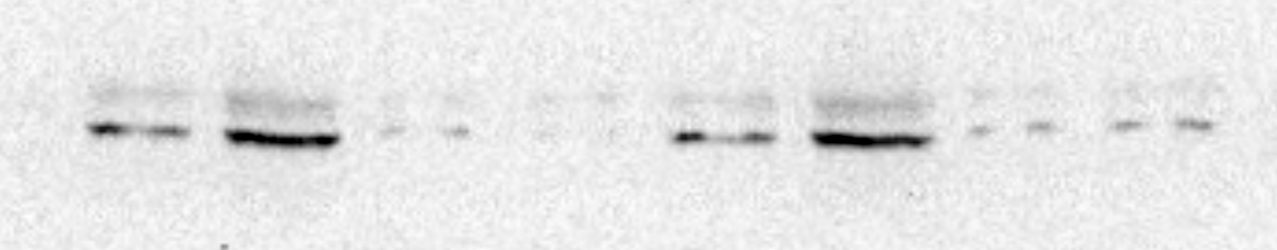


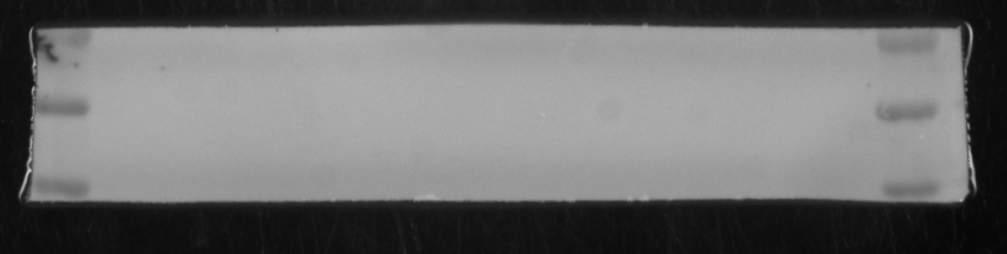


70kD

55kD

43kD

**UCP1**


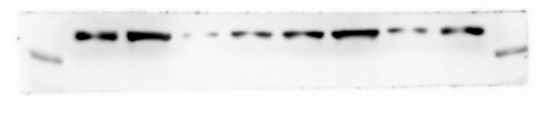


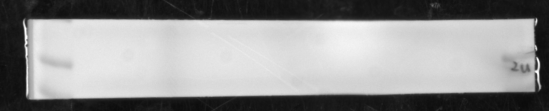


34kD

25kD

15kD

**PGC1A**


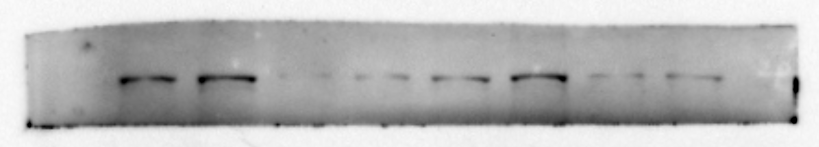


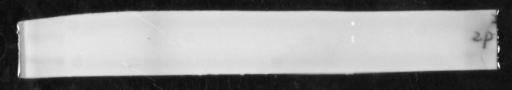


130kD

100kD

**CIDEA**


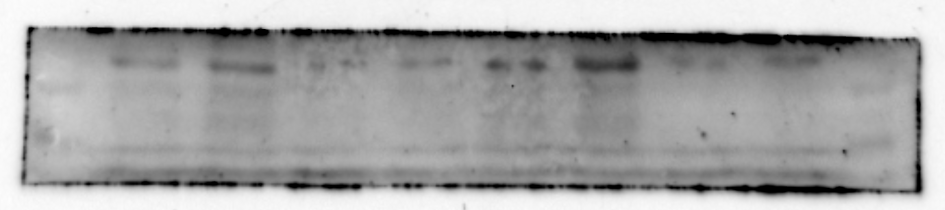


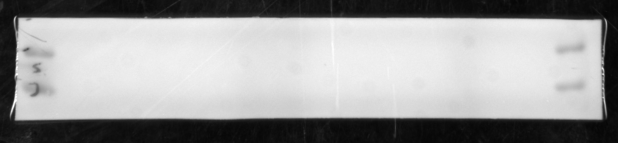


25kD

15kD

**DIO2**


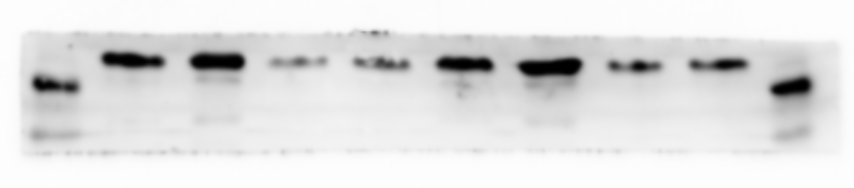


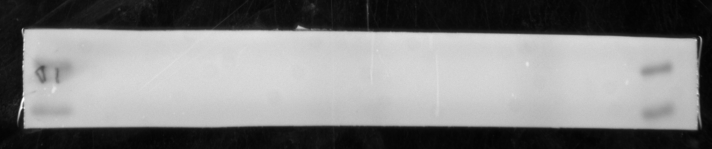


25kD

15kD

**β-actin**


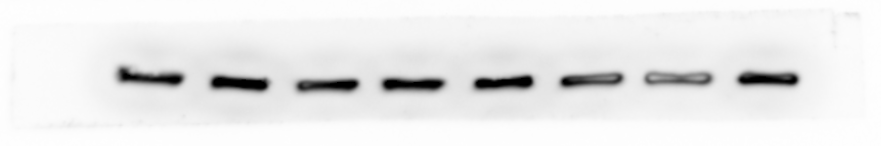


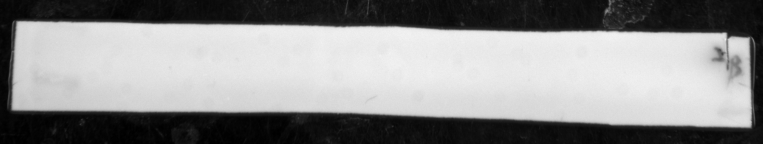


55kD

43kD

**Fig. 7F**

**KLF12**


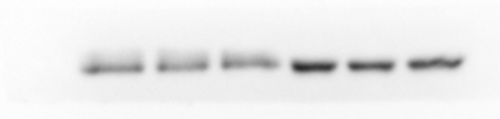


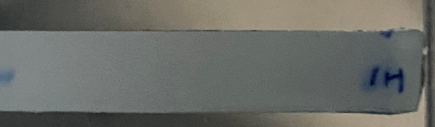


55kD

43kD

**UCP1**


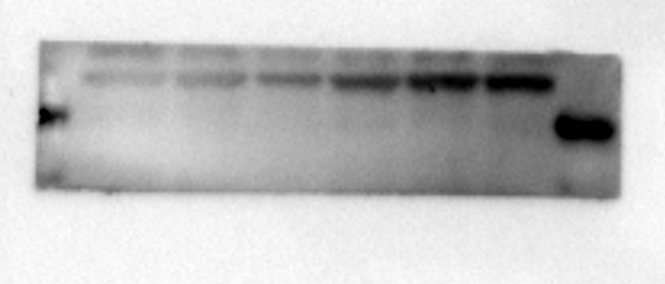


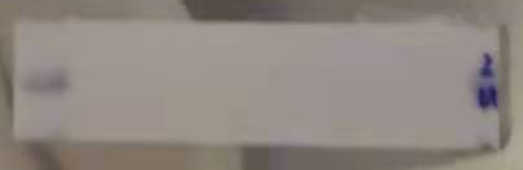


34kD

25kD

**PGC1A**


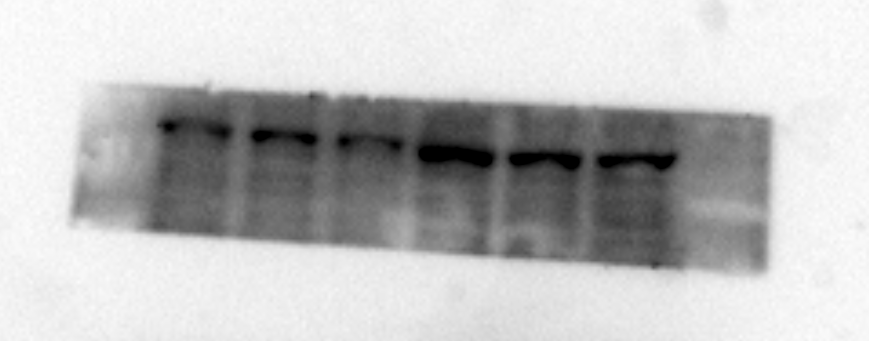


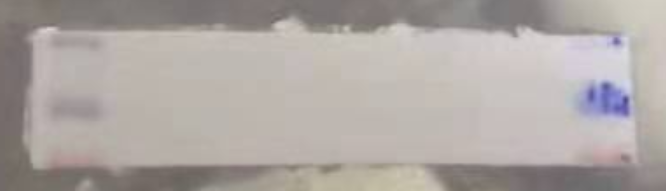


130kD

100kD

70kD

**CIDEA**


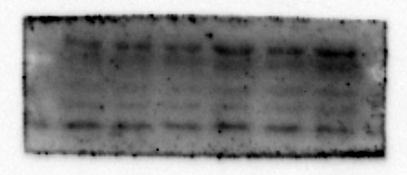


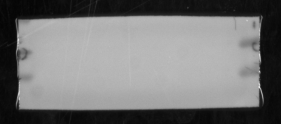


34kD

25kD

15kD

**DIO2**


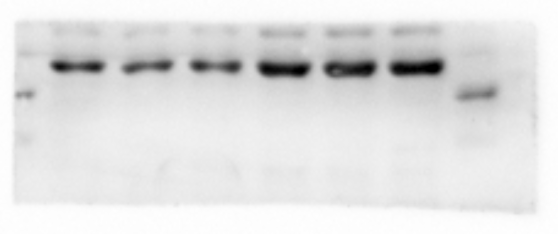


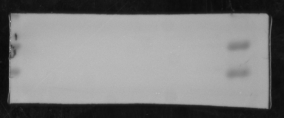


34kD

25kD

15kD

**β-actin**


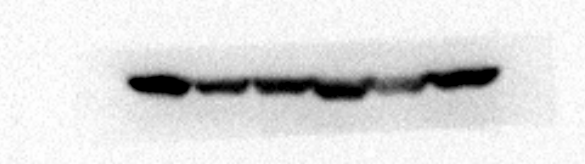


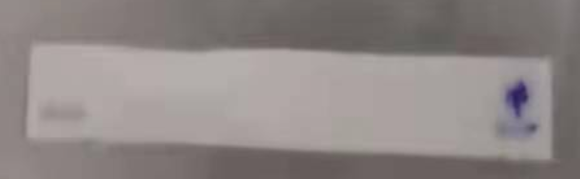


55kD

43kD

**Supplementary Figure 4C**

**COL18A1**


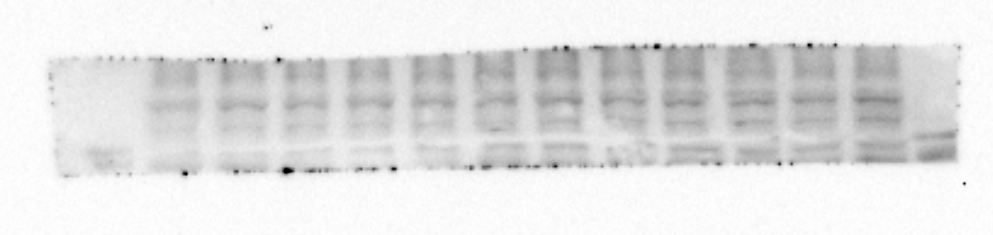


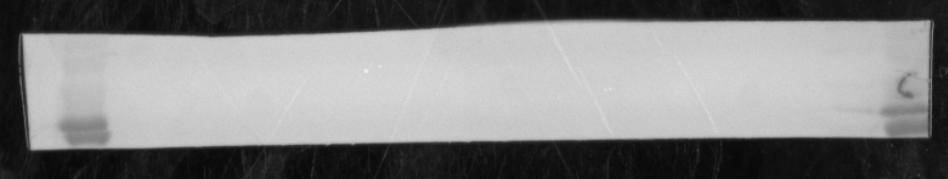


300kD

250kD

180kD

**β-actin**


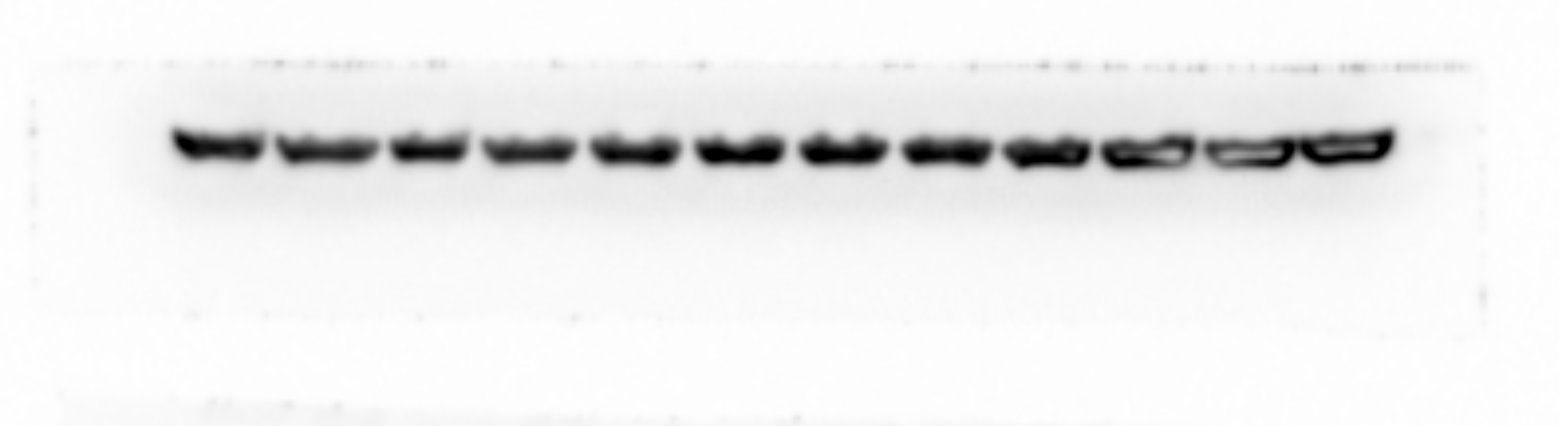


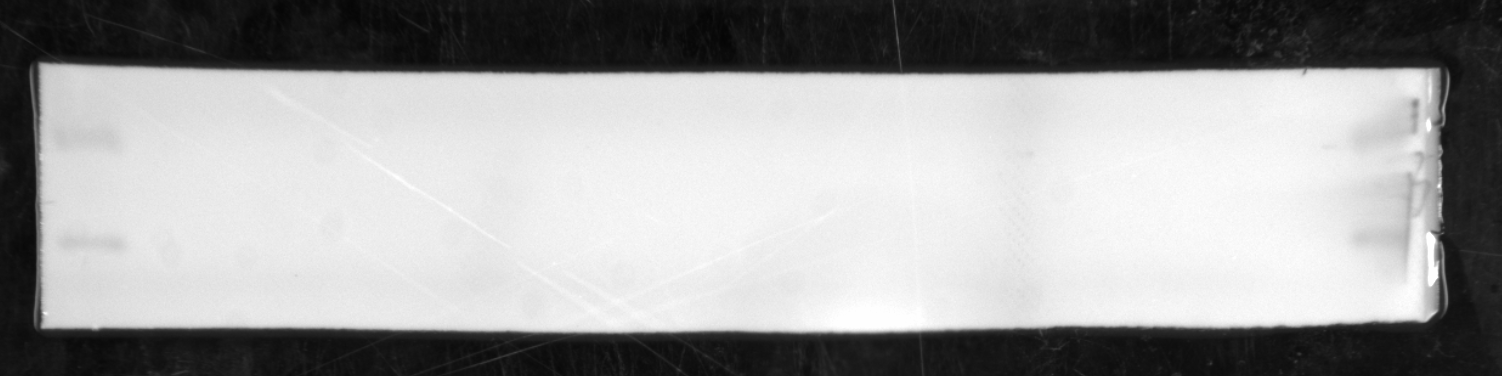


43kD

34Kd

**Supplementary Figure 8A**

**KLF12**


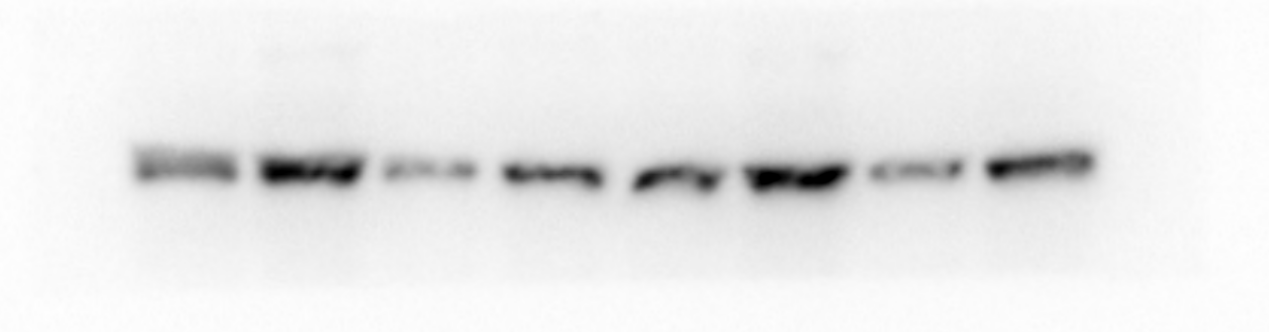


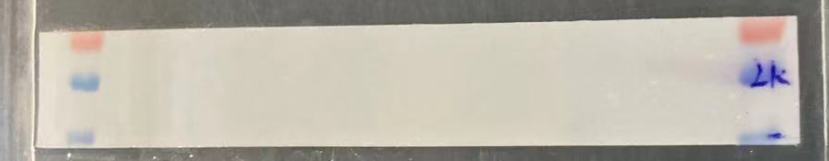


70kD

55kD

43kD

**β-actin**


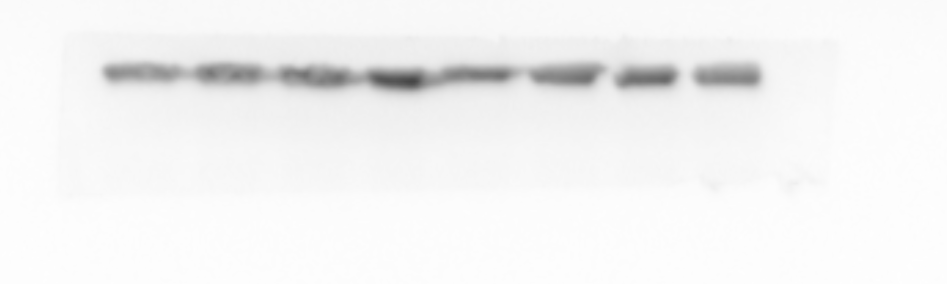


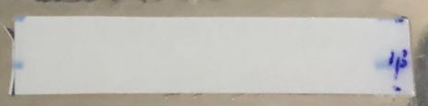


55kD

43kD
